# Construction and multi-center validation of a model based on blending ensemble learning for predicting stroke-associated pneumonia following acute ischemic stroke

**DOI:** 10.3389/fneur.2026.1841730

**Published:** 2026-09-18

**Authors:** Xinying Liu, Qiang Fu, Shuo Yang, Lin Zhang, Boxuan Feng, Ying Gao, Chengboya Zhao, Di Gao, Guanwei Fan, Li Liu, Xiaokun Yang

**Affiliations:** 1 First Teaching Hospital of Tianjin University of Traditional Chinese Medicine, Tianjin, China; 2 National Clinical Research Center for Chinese Medicine, Tianjin, China

**Keywords:** acute ischemic stroke, blending ensemble learning, machine learning, risk stratification, stroke-associated pneumonia

## Abstract

**Background:**

Stroke-associated pneumonia (SAP) is a common and serious infectious complication following acute ischemic stroke (AIS), leading to worsened clinical outcomes. We aimed to develop and validate a novel predictive model based on Blending Ensemble Learning, to enable early, precise screening and individualized risk stratification for SAP.

**Methods:**

In this multi-center study, a derivation cohort was initially established using 1,123 patients admitted between 2022 and 2024. Subsequently, 199 patients were prospectively enrolled in early 2025 as a temporal validation set, and 287 patients were extracted from a regional health big data platform (covering 12 hospitals) to serve as an external validation set. High-value clinical and biomarker features were identified via least absolute shrinkage and selection operator (LASSO) regression. To overcome the limitations of single classifiers, a Blending Ensemble Learning strategy was adopted to construct the final predictive model. The model underwent comprehensive evaluation for discrimination, calibration, and clinical utility, and was benchmarked against single random forest (RF) and extreme gradient boosting (XGBoost) models.

**Results:**

Seventeen predictors were identified, including systemic immune-inflammation index (SII), neutrophil-to-lymphocyte ratio (NLR), the National Institutes of Health Stroke Scale (NIHSS), and D-dimer. The Blending ensemble model demonstrated superior discrimination in the validation cohort, achieving an Area Under the Receiver Operating Characteristic Curve (AUC) of 0.898 (95% CI: 0.845–0.951), which significantly outperformed the optimal single model. Furthermore, SHapley Additive exPlanations (SHAP) analysis highlighted hyperinflammation, immunothrombosis, and nutritional depletion as pivotal mechanisms driving SAP pathogenesis.

**Conclusion:**

The proposed Blending ensemble model exhibits excellent generalizability and high sensitivity. The accompanying web-based tool provides robust decision support for identifying high-risk patients and implementing early precision interventions.

## 1 Introduction

Acute ischemic stroke (AIS) remains a leading cause of mortality and long-term disability globally (1). Despite significant advancements in acute reperfusion strategies, such as intravenous thrombolysis and endovascular treatment, post-stroke complications continue to severely impede neurological recovery and impose a substantial healthcare burden (2). Notably, Stroke-Associated Pneumonia (SAP), recognized as the most prevalent infectious complication following AIS, has a reported incidence ranging from 7 to 38% across various cohort studies (3). SAP is not only associated with prolonged hospital stays and increased demand for intensive care but serves as an independent risk factor for early mortality and poor long-term outcomes (4). Consequently, the early and precise identification of patients at high risk for SAP is of paramount importance for guiding preventive interventions and improving clinical prognoses.

Currently, several risk stratification tools, such as the A2DS2 and ISAN, have been developed for SAP screening (5). However, these scales are predominantly constructed using traditional logistic regression models and rely heavily on static demographic and baseline clinical variables. Consequently, they often fail to capture dynamic pathophysiological changes in biomarkers, resulting in suboptimal predictive sensitivity and specificity (6). Emerging evidence suggests that the interplay between “Stroke-induced Immunodepression (SIID)” and “Immunothrombosis” constitutes a pivotal mechanism driving the pathogenesis of SAP (7). Compared to single indicators such as white blood cell (WBC) or C-reactive protein (CRP), novel systemic inflammatory composite indices offer a more comprehensive reflection of the balance between the body’s immune homeostasis and thromboinflammatory response. These indices, which include the systemic immune-inflammation index (SII), the neutrophil‑to‑lymphocyte ratio (NLR), and the Systemic Inflammation Response Index (SIRI), are regarded as highly promising predictive biomarkers (8, 9). Nevertheless, there remains a paucity of research integrating these robust indices into high-precision predictive models.

In recent years, Machine Learning (ML) algorithms have demonstrated performance superior to conventional statistical models in predicting post-stroke hemorrhagic transformation, recurrence, and functional outcomes (10). Despite preliminary attempts to apply ML for SAP prediction, existing models remain constrained by substantial methodological limitations. Specifically, model architectures are predominantly confined to single algorithms, and there is a pervasive absence of rigorous temporal validation and independent external multi-center validation. These deficiencies severely compromise the generalizability and clinical utility of current predictive tools (11).

To address these challenges, this study aimed to construct and validate a high-performance ML risk model for SAP. Specifically, we implemented a multi-faceted approach to improve model performance and generalizability: (1) incorporating multidimensional predictors, particularly composite indices such as the SII, NLR, and SIRI, which reflect the SIID mechanism; (2) employing a Blending Ensemble Strategy to specifically optimize diverse base learners for enhanced robustness; and (3) enforcing a rigorous “spatio-temporal dual validation” framework with independent and external multi-center cohorts, while ensuring clinical accessibility through an interpretable web-based tool using Shapley additive explanations (SHAP). Ultimately, this study seeks to provide a quantitative and accessible SAP risk screening instrument for acute stroke patients, thereby supporting early clinical intervention and improving patient outcomes.

## 2 Methods

### 2.1 Data source

We used a two-way cohort comparison design consisting of one derivation cohort and two independent validation cohorts to rigorously evaluate the model’s performance across multiple dimensions. This study was approved by the Ethics Committee of the First Teaching Hospital of Tianjin University of Traditional Chinese Medicine (No. TYLL2025[Z]044). Given that this was a non-interventional observational study utilizing anonymized clinical data, the requirement for informed consent was waived by the Ethics Committee.

#### 2.1.1 Derivation cohort

This study consecutively enrolled patients diagnosed with AIS who were admitted to the First Teaching Hospital of Tianjin University of Traditional Chinese Medicine between January 1, 2022, and December 31, 2024. Based on the clinical outcome of developing SAP during hospitalization, the study population was stratified into the SAP group and the Non-SAP group. Informed consent was waived due to the retrospective nature of the study and the use of anonymized data.

#### 2.1.2 Temporal validation cohort

A temporal validation cohort was established to evaluate the model’s stability over time, incorporating patients admitted to the same institution between January 1, 2025, and June 30, 2025. This cohort acted as a prospective simulation to detect potential “temporal drift” in predictive performance.

#### 2.1.3 External multi-center validation cohort

External validation was performed using a multi-center cohort from the Inspur Health Database (Tianjin, 12 medical centers). A total of 300 AIS patients (2022–2024) were selected via stratified random sampling. Inclusion and exclusion criteria were strictly harmonized with the derivation cohort to ensure comparability.

### 2.2 Eligibility criteria

(1) Age: Aged between 18 and 85 years. (2) Diagnosis: A primary diagnosis of AIS in accordance with the Chinese guidelines for diagnosis and treatment of acute ischemic stroke 2023, confirmed by definitive neuroimaging (brain CT or MRI) (12). (3) Admission Window: Admitted to the hospital within 24 h of symptom onset.

### 2.3 Excluded criteria

(1) Pre-existing Infection: Evidence of active infection upon admission, such as elevated body temperature exceeding 38.0 °C or the presence of pulmonary infiltrates on chest imaging, or a confirmed history of infection or antibiotic use within the preceding 2 weeks, was required to strictly exclude Community-Acquired Pneumonia or other latent infections; (2) Severe Comorbidities: Presence of severe life-limiting conditions, including advanced malignancy, end-stage renal disease, severe hepatic failure, or uncontrolled congestive heart failure; (3) Insufficient Observation Window: Patients who died or were discharged (including discharge against medical advice) within 72 h of admission, precluding a definitive assessment of the SAP outcome; (4) Data Unavailability: Missing primary outcome data (SAP status) or a missing data rate exceeding 20% for candidate predictors.

### 2.4 Outcome definition

The primary outcome was the in-hospital incidence of SAP, defined according to the modified Centers for Disease Control and Prevention criteria (13). At least 1 of the following: 1. Fever (>38°C) with no other recognized cause; 2. Leukopenia (<4,000 WBC/mm^3^) or leukocytosis (>12,000 WBC/mm^3^); 3. For adults ≥70 years old, altered mental status with no other recognized cause. And at least 2 of the following:1. New onset of purulent sputum, or change in character of sputum over a 24 h period, or increased respiratory secretions, or increased suctioning requirements; 2. New onset or worsening cough, or dyspnea, or tachypnea (respiratory rate>25/min); 3. Rales, crackles, or bronchial breath sounds; 4. Worsening gas exchange (e.g., O_2_ desaturation [e.g., PaO_2_/FiO_2_ ≤ 240], increased oxygen requirements). And ≥2 serial chest radiographs with at least 1 of the following: New or progressive and persistent infiltrate, consolidation, or cavitation. To minimize assessment bias and establish a robust “Ground Truth,” the diagnosis of SAP was independently adjudicated by two senior clinicians who were blinded to the predictor variables. Any discrepancies were adjudicated by a third independent reviewer.

### 2.5 Data collection

This study systematically extracted multidimensional baseline clinical data within 24 h of admission. Regarding demographic and clinical profiles, the collected variables comprised age, sex, specific marital status, lifestyle habits such as smoking and alcohol consumption, and major comorbidities, notably hypertension, diabetes, atrial fibrillation (FIB), and chronic obstructive pulmonary disease (COPD). Stroke-specific metrics comprised the admission the National Institutes of Health Stroke Scale (NIHSS), stroke subtype, and functional swallowing status. Dysphagia was systematically screened within 24 h of admission using the standardized bedside Kubota Water Swallow Test (KWST) administered by trained stroke nurses before any oral intake. A KWST grade of ≥3 was defined as the presence of dysphagia. In terms of laboratory parameters, beyond routine complete blood counts, coagulation profiles, and comprehensive biochemistry (liver/renal function, glucose, lipids, electrolytes), this study specifically prioritized the assessment of the immuno-inflammatory state. We incorporated not only traditional markers (CRP, hs-CRP) but also novel composite inflammatory indices, including the NLR, SII, and SIRI.

### 2.6 Data preprocessing and feature selection

The derivation cohort (*n* = 1,123) was partitioned into a development set (80%, *n* = 898) and a held-out internal test set (20%, *n* = 225) before any model training, aiming to construct a robust and generalizable predictive model. Within the development set, a rigorous stratified 5-fold cross-validation (CV) framework was implemented. To rigorously prevent data leakage and eliminate “optimistic bias,” all data preprocessing and feature selection procedures were strictly restricted within the training folds of each CV iteration.

Initially, the missingness mechanism was assumed to be Missing at Random based on clinical routine testing patterns. Candidate variables exhibited low overall missingness (all < 20%). Missing data were subsequently imputed using the MissForest algorithm, a random forest (RF)-based non-parametric method. The imputation model was fitted exclusively on the training set (80% of data) and subsequently applied to the corresponding test set (20%), thereby preserving the independence of validation subsets. Furthermore, a sensitivity analysis was performed on complete cases across both the internal test set and the temporal validation cohort to assess the robustness against potential imputation-induced bias. Subsequently, to address class imbalance, the Synthetic Minority Over-sampling Technique was applied within the training set to equilibrate the ratio between SAP and Non-SAP groups. Conversely, the test set retained its original distribution to objectively evaluate the model’s discriminatory efficacy in real-world clinical scenarios.

Feature selection was executed in a two-stage process. First, univariate analysis was employed to eliminate redundant variables (*p* > 0.05), serving as a preliminary filtration to reduce computational noise and dimensionality prior to regularization. Second, least absolute shrinkage and selection operator (LASSO) regression was introduced for high-dimensional dimensionality reduction. To guarantee the robustness of predictors against data perturbations, we established strict “Stability Selection Criteria”: only features retained by the LASSO algorithm across all 5-fold CV iterations (selection frequency = 5/5) were included. This “intersection strategy” ensured that the associations between selected features and the outcome were stable and consistent across diverse data distributions. Finally, to mitigate potential multicollinearity among the selected predictors, we performed a collinearity diagnostic using the variance inflation factor (VIF). Features with a VIF > 5 were considered to exhibit significant redundancy and were systematically excluded to ensure the stability and interpretability of the final model.

### 2.7 Model construction and statistical evaluation

All algorithmic development and statistical analyses were conducted in a Python 3.9 environment. To comprehensively assess the efficacy of diverse ML paradigms in SAP prediction, we constructed and systematically benchmarked nine representative supervised learning algorithms, spanning four distinct categories: (1) Ensemble tree models: Extreme Gradient Boosting (XGBoost), Light Gradient Boosting Machine (LightGBM), Gradient Boosting Decision Tree (GBDT), RF, and Adaptive Boosting (AdaBoost); (2) Basic tree model: Decision Tree (DT); (3) Kernel-based and distance-based algorithms: Support Vector Machine (SVM) and K-Nearest Neighbors (KNN); and (4) Probabilistic model: Gaussian Naive Bayes (GNB).

Stratified 5-fold CV served as the primary strategy to bolster model generalizability and counteract overfitting, ensuring that class distributions remained consistent across all training and validation subsets. Within this framework, Grid Search was utilized for the systematic, fine-grained optimization of key hyperparameters (Supplementary Table S1). To guarantee the strict reproducibility of all computational results, a fixed random seed of 40 was applied throughout the entire modeling process. Model performance was evaluated using a multidimensional metric system, including the Area Under the Receiver Operating Characteristic Curve (AUC), accuracy, sensitivity, specificity, Positive Predictive Value (PPV), Negative Predictive Value (NPV), and *F*1-score. 95% Confidence Intervals (CIs) for these metrics were estimated via bootstrap resampling. Differences in AUC were compared using the DeLong test. A Bonferroni-adjusted *p* < 0.0056 was defined as the threshold for statistical significance, accounting for multiple comparisons across nine models.

### 2.8 Construction of the blending ensemble strategy

To transcend the performance limitations of single models and further enhance the generalizability and variance reduction of the predictive system, we constructed a Blending Ensemble Strategy based on a two-layer architecture. The core rationale of this strategy lies in leveraging the intrinsic heterogeneity of distinct algorithms to achieve synergistic complementary advantages.

Phase 1: Based on the performance benchmarking from the aforementioned stratified 5-fold CV, and adhering to the principle of prioritizing both “high performance and diversity,” we strategically selected XGBoost and RF from the nine candidate algorithms as the Level-1 Base Learners. These models represent two mainstream ensemble paradigms, such as Gradient Boosting and Bagging, thereby enabling the capture of data features from multidimensional perspectives and ensuring the structural diversity of the ensemble system.

Phase 2: To ensure a rigorous evaluation, the retrospective population was initially bifurcated into a development set and a completely independent internal test set. Within the development set, a 70/30 random split was implemented to create a training subset and a hold-out subset, specifically designed to facilitate the two-layer blending architecture.

The Level-1 base learners were trained on the training subset (70%). Subsequently, these trained models were used to generate prediction probability vectors for the hold-out subset (30%). These vectors were reconstructed into a meta-feature matrix, serving as the exclusive input for the Level-2 logistic regression meta-learner.

Through this hierarchical isolation, the meta-learner was trained on “unseen” data, enabling the system to calibrate optimal integration weights based on generalized performance rather than memorized training outcomes. This multi-stage data safeguarding effectively mitigated overfitting and data leakage before the final model was frozen and validated on the independent internal test set. The systemic workflow, encompassing initial data partitioning, nested feature selection, and the hierarchical training of base and meta-learners, is explicitly delineated in Table 1.

**Table 1:** Strictly nested blending implementation and data isolation.

Input: total retrospective cohort
Step 1: initial data partitioning
Split data into Development (80%) and Independent Test (20%) sets.
Step 2: nested feature selection
Perform VIF analysis solely on the Development set.
Exclude redundant features.
Data Safeguard: Selection parameters were estimated solely from training folds.
Step 3: two-tier blending architecture construction
Sub-partitioning: Split Development Set into Training (70%) and Hold-out (30%).
Level-1: Train RF and XGBoost on Training Subset using stratified 5-fold CV.
Level-2: Apply Level-1 models to Hold-out Subset to generate probability vectors, then train a Logistic Regression meta-learner to determine optimal fusion weights.
Step 4: final evaluation
Evaluate the frozen model on the Independent Test set.

CV, cross-validation; RF, random forest; XGBoost, extreme gradient boosting; VIF, variance inflation factor.

### 2.9 Performance comparison with established clinical risk scores

For rigorous evaluation of the clinical incremental value of the proposed Blending ensemble model, we performed a head-to-head comparison with the widely used A2DS2 clinical risk scale. Specifically, the A2DS2 was calculated for each patient in the validation cohorts based on its original weighting criteria. The DeLong test was then employed to determine whether the difference in AUC between the final Blending model and the A2DS2 reached statistical significance. Furthermore, to quantify the improvement in risk reclassification, the Net Reclassification Index (NRI) and Integrated Discrimination Improvement (IDI) were calculated.

### 2.10 Model interpretability and visualization

The complex internal structure of ML often leads to an inherent “black-box” opacity, which hinders its trustworthiness and translation into clinical practice. To address this challenge and enhance model transparency, we introduced the SHAP framework. Based on the validation dataset, SHAP values were calculated for each feature in the optimal model to precisely quantify the marginal contribution of individual predictors to the probability of SAP occurrence.

The interpretability analysis was conducted across two dimensions. First, for Global Interpretability, we utilized the SHAP Summary Beeswarm Plot to intuitively visualize the ranking of feature importance and the direction of their impact. In this plot, each dot represents a single sample. The color gradient (red/blue) indicates the magnitude of the feature value, while the horizontal position reveals whether the feature exerts a positive contribution or negative suppression on the model’s output log-odds. Second, for Local Interpretability, we employed the SHAP Force Plot to perform a case-level micro-analysis of representative patients. This visualization tool deconstructs the prediction for a specific individual, demonstrating how key variables act synergistically to propel the risk of SAP toward a higher or lower probability. Such individual-level visualization facilitates clinicians in rapidly identifying patient-specific drivers of risk, thereby supporting precision decision-making.

### 2.11 Assessment of model generalizability

To rigorously evaluate the generalizability and robustness of the final Blending model, we implemented a dual-dimension validation strategy. First, temporal validation was conducted to assess the model’s stability against “temporal drift,” ensuring that the predictive performance remains consistent despite potential shifts in clinical practice patterns over time. Second, external multi-center validation was performed to verify the model’s geographical transportability. By testing the algorithm on data from 12 distinct medical centers, we aimed to confirm its ability to handle inter-center heterogeneity and its readiness for broad clinical deployment.

### 2.12 Statistical analysis

Statistical analyses of clinical baseline characteristics were performed using SPSS software, version 26.0. Initially, the normality of continuous variables was evaluated using the Shapiro–Wilk test in conjunction with the visual inspection of histograms. Continuous variables conforming to a normal distribution were expressed as mean ± standard deviation and compared using the independent samples t-test. Non-normally distributed variables were described as median (interquartile range [IQR]) and analyzed via the Mann–Whitney U test. Categorical variables were presented as frequencies and percentages [n (%)] and compared using Pearson’s χ^2^ test or Fisher’s exact test, as appropriate based on expected counts. All statistical tests were two-sided, and *p* ≤ 0.05 was considered statistically significant. To mitigate the risk of Type I errors associated with multiple comparisons of baseline variables, *p*-values were adjusted using the Benjamini-Hochberg False Discovery Rate method.

## 3 Results

### 3.1 Study population and baseline

Characteristics A total of 1,187 patients with AIS admitted between January 1, 2022, and December 31, 2024, were initially screened. Based on strict inclusion and exclusion criteria, 64 patients were disqualified for the following reasons: pre-existing infection prior to admission (*n* = 34), severe life-limiting comorbidities or malignancy (*n* = 12), length of hospital stay < 72 h (*n* = 10), and missing key clinical data (*n* = 8). Ultimately, 1,123 patients were included in the Derivation Cohort. Among them, 236 (21.02%) were diagnosed with SAP (SAP group), while the remaining 887 were classified into the Non-SAP group. To validate the robustness of the model, two independent validation cohorts were established. The temporal validation cohort prospectively screened 217 patients from the Emergency Department of the First Teaching Hospital of Tianjin University of Traditional Chinese Medicine (January to June 2025). After excluding 18 patients (16 with pre-existing infections and 2 with severe comorbidities), 199 were finally included. The external validation cohort employed a time-stratified sampling strategy to initially select 300 cases from 12 medical centers in Tianjin (2022–2024) covered by the Inspur Health Database. Following the exclusion of 13 patients (3 with infections and 10 with missing key data), 287 were finally enrolled. The detailed flowchart of patient recruitment is illustrated in Figure 1.

**Figure 1:**
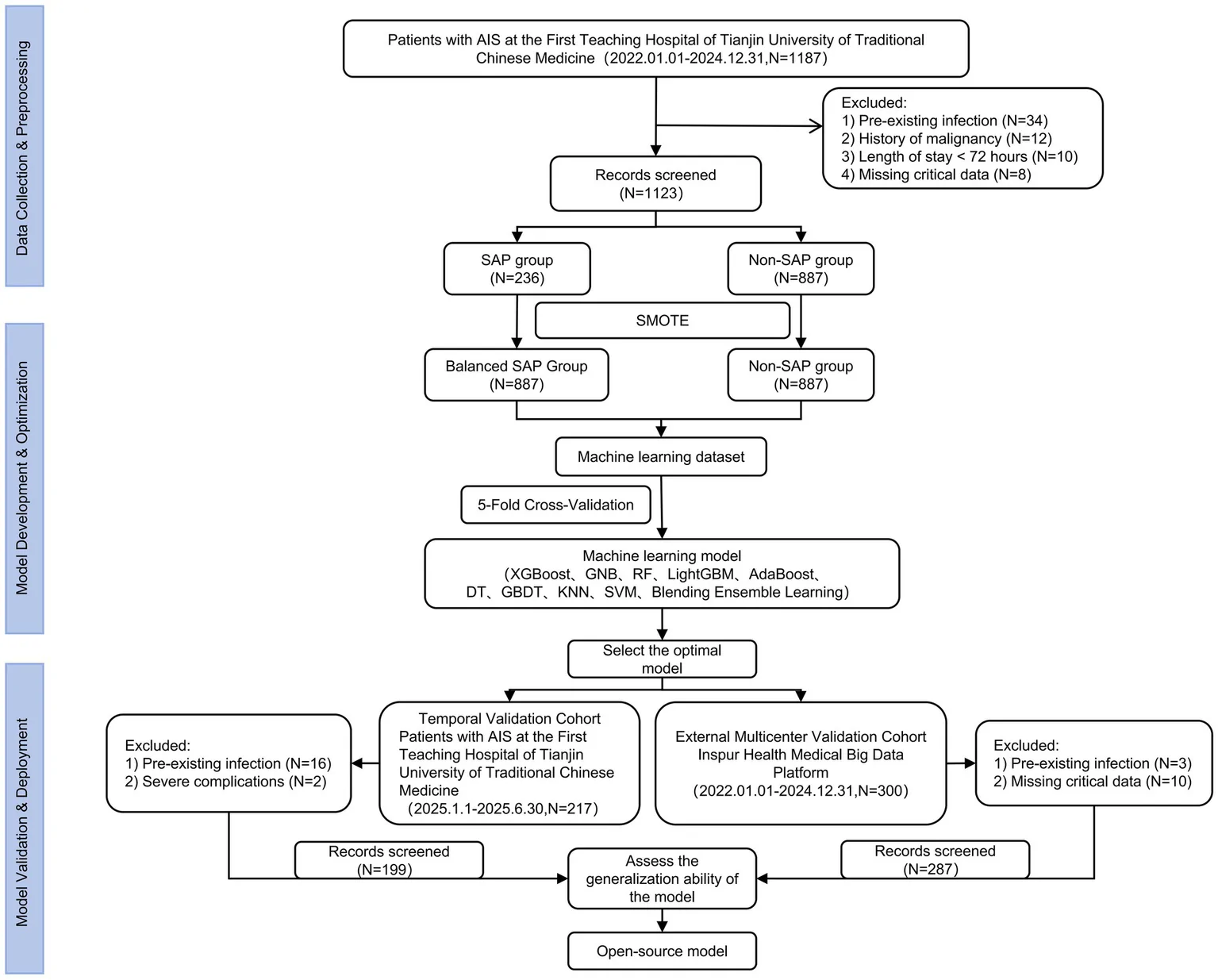
Flowchart of the study design and patient selection. The diagram illustrates the systematic workflow encompassing data collection, feature engineering, model development, and multi-dimensional validation across three cohorts.

The baseline demographic and clinical characteristics of the derivation cohort are summarized in Supplementary Table S2. Univariate analysis revealed significant disparities between the SAP and Non-SAP groups across multiple dimensions. Regarding demographics and clinical presentation, patients in the SAP group were significantly older (*p* < 0.001) and exhibited greater disease severity upon admission, evidenced by higher baseline NIHSS (*p* < 0.001) and a higher prevalence of dysphagia. In terms of laboratory parameters, the SAP group demonstrated a state of pronounced systemic inflammation. Compared to the Non-SAP group, levels of leukocyte count, absolute neutrophil count, and CRP were markedly elevated. Notably, the novel composite inflammatory indices prioritized in this study, such as the NLR, Platelet-to-Lymphocyte Ratio (PLR), SIRI, and SII, were all significantly elevated in the SAP group (*p* < 0.001). Conversely, serum albumin (ALB) and hemoglobin (HGB) levels were significantly lower in the SAP group, suggesting a poorer nutritional status in these patients.

### 3.2 Feature selection results

During the feature space construction phase, all evaluated candidate variables satisfied the data completeness criterion, with none exceeding the pre-specified 20% missing threshold, and detailed missing distributions are reported in Supplementary Table S1. Subsequently, for the candidate features that passed univariate preliminary screening (*p* < 0.05), missing values were imputed using the MissForest algorithm, which is an iterative imputation method based on RF. This approach effectively preserves the complex non-linear structures inherent in the data.

To mitigate the issue of class imbalance present in the original training sets, the Synthetic Minority Over-sampling Technique was applied for resampling optimization. Following this standardized protocol, the ratio of positive to negative samples within each training fold was adjusted to a balanced 1:1 equilibrium.

We rigorously executed a “two-stage feature selection strategy” to identify the most robust predictors for SAP. Initially, 45 potential candidate variables were preliminarily screened from the original high-dimensional data via univariate analysis (*p* < 0.05). Subsequently, these candidate features were subjected to deep dimensionality reduction using the LASSO regression model nested within the 5-fold CV framework.

To guarantee the model’s generalizability across diverse data distributions, a strict “Intersection-based Stability Selection Criterion” was enforced: only variables that consistently exhibited non-zero coefficients across all 5-fold CV were retained (Supplementary Table S3). Based on this criterion, 18 pivotal predictors were ultimately finalized, encompassing: (1) Neurological and clinical status metrics: Age, NIHSS, modified Glasgow Prognostic Score (mGPS), A2DS2, dysphagia, stroke subtype, COPD, and smoking history; (2) Pivotal immuno-inflammatory markers: SII, NLR, and hs-CRP; (3) Fundamental physiological and coagulation-nutritional indices: WBC, platelet count (PLT), HGB, mean corpuscular hemoglobin concentration (MCHC), ALB, FIB, and D-dimer.

It is noteworthy that, despite SIRI and PLR demonstrating statistical significance in the univariate analysis, they were excluded due to coefficient instability during the multi-fold LASSO validation.

Furthermore, collinearity diagnostics were performed using the VIF to evaluate potential multicollinearity among the 18 candidate variables. The results indicated that the A2DS2 exhibited severe multicollinearity (VIF = 7.42) (Supplementary Table S4), attributed to its structural redundancy with Age, NIHSS, and dysphagia. To ensure the robustness and stability of the final Blending Ensemble model, the A2DS2 was excluded, resulting in a finalized set of 17 pivotal predictors for model construction.

### 3.3 Multi-model construction and performance

Based on the 17 identified pivotal features, nine ML classifiers employing distinct algorithmic mechanisms were constructed and trained. The detailed performance metrics of each model on the independent validation set are summarized in Table 2.

**Table 2:** Predictive performance of nine machine learning algorithms for stroke-associated pneumonia in the validation cohort.

Model^a^	AUC (95% CI)	Cutoff (95% CI)	Accuracy (95% CI)	Sensitivity (95% CI)	Specificity (95% CI)	PPV (95% CI)	NPV (95% CI)	*F*1 Score (95% CI)	Kappa (95% CI)
RF	0.811 (0.730–0.892)	0.646(0.620–0.672)	0.708(0.685–0.730)	0.659(0.623–0.695)	0.79(0.725–0.855)	0.845(0.810–0.880)	0.577(0.555–0.598)	0.739(0.718–0.761)	0.417(0.370–0.464)
XGBoost	0.800 (0.716–0.884)	0.648(0.593–0.703)	0.743(0.706–0.780)	0.743(0.710–0.776)	0.743(0.685–0.801)	0.832(0.798–0.866)	0.629(0.586–0.673)	0.785(0.754–0.815)	0.468(0.392–0.545)
AdaBoost	0.760 (0.667–0.852)	0.506(0.504–0.509)	0.711(0.699–0.724)	0.707(0.669–0.745)	0.719(0.649–0.789)	0.814(0.784–0.843)	0.591(0.574–0.608)	0.755(0.742–0.768)	0.407(0.374–0.440)
GBDT	0.746 (0.649–0.842)	0.964(0.902–1.026)	0.701(0.676–0.726)	0.695(0.611–0.780)	0.71(0.620–0.799)	0.808(0.773–0.843)	0.587(0.536–0.637)	0.743(0.706–0.779)	0.387(0.355–0.420)
KNN	0.724 (0.626–0.821)	1.0(1.000–1.000)	0.523(0.506–0.540)	0.293(0.269–0.317)	0.914(0.896–0.933)	0.854(0.828–0.880)	0.432(0.421–0.442)	0.436(0.408–0.464)	0.169(0.144–0.193)
GNB	0.710 (0.612–0.809)	0.026(0.014–0.039)	0.678(0.658–0.698)	0.735(0.705–0.764)	0.581(0.527–0.635)	0.75(0.729–0.771)	0.563(0.534–0.592)	0.742(0.726–0.758)	0.313(0.267–0.359)
DT	0.697 (0.600–0.793)	0.659(0.646–0.672)	0.666(0.606–0.725)	0.682(0.619–0.744)	0.638(0.578–0.698)	0.761(0.715–0.807)	0.543(0.475–0.612)	0.719(0.664–0.773)	0.309(0.193–0.425)
LightGBM	0.604 (0.513–0.695)	0.631(0.630–0.631)	0.593(0.541–0.646)	0.592(0.437–0.748)	0.595(0.432–0.758)	0.723(0.672–0.774)	0.477(0.408–0.545)	0.636(0.552–0.721)	0.178(0.103–0.253)
SVM	0.548 (0.439–0.656)	0.716(0.653–0.779)	0.507(0.457–0.558)	0.388(0.216–0.559)	0.71(0.511–0.908)	0.713(0.653–0.772)	0.403(0.375–0.432)	0.475(0.344–0.606)	0.083(0.014–0.152)

AUC, area under the receiver operating characteristic curve; CI, confidence interval; PPV, positive predictive value; NPV, negative predictive value; RF, random forest; XGBoost, extreme gradient boosting; AdaBoost, adaptive boosting; GBDT, gradient boosting decision tree; GNB, Gaussian Naive Bayes; DT: decision tree; KNN, k-nearest neighbors; SVM, support vector machine; LightGBM, light gradient boosting machine. ^a^Models are ranked by AUC values in descending order. Data are presented as mean (95% CI).

In the benchmarking of candidate algorithms, Ensemble Tree Models consistently exhibited superior discriminatory efficacy compared to distance-based or linear models. Among the nine base learners, the RF model demonstrated the most robust comprehensive performance, achieving the highest AUC of 0.811 (95% CI: 0.730–0.892), while maintaining a well-balanced predictive profile with an *F*1-score of 0.739 and a Sensitivity of 0.659 (Table 2). XGBoost followed closely as the second-best performer with an AUC of 0.800 (95% CI: 0.716–0.884) and a high Average Precision (AP) of 0.868. Other ensemble methods, such as AdaBoost (AUC = 0.760) and GBDT (AUC = 0.746), also showed stable predictive capabilities. In contrast, models based on linear segmentation or simpler heuristics, such as SVM (AUC = 0.548) and LightGBM (AUC = 0.604), exhibited relatively modest performance on this dataset.

The performance disparities were further visualized through multi-dimensional evaluation curves (Figure 2). The Receiver Operating Characteristic (ROC) curves (Figure 2A) and Precision-Recall (PR) curves (Figure 2B) illustrated that the RF model possessed the superior area under the curve (AUC = 0.811) and the highest AP of 0.869 (95% CI: 0.837–0.901). Furthermore, Calibration Curves (Figure 2D) indicated that the RF model yielded the best probabilistic calibration with the lowest Brier score of 0.174 (95% CI: 0.163–0.186), demonstrating a high degree of agreement between predicted SAP risks and observed clinical outcomes.

**Figure 2:**
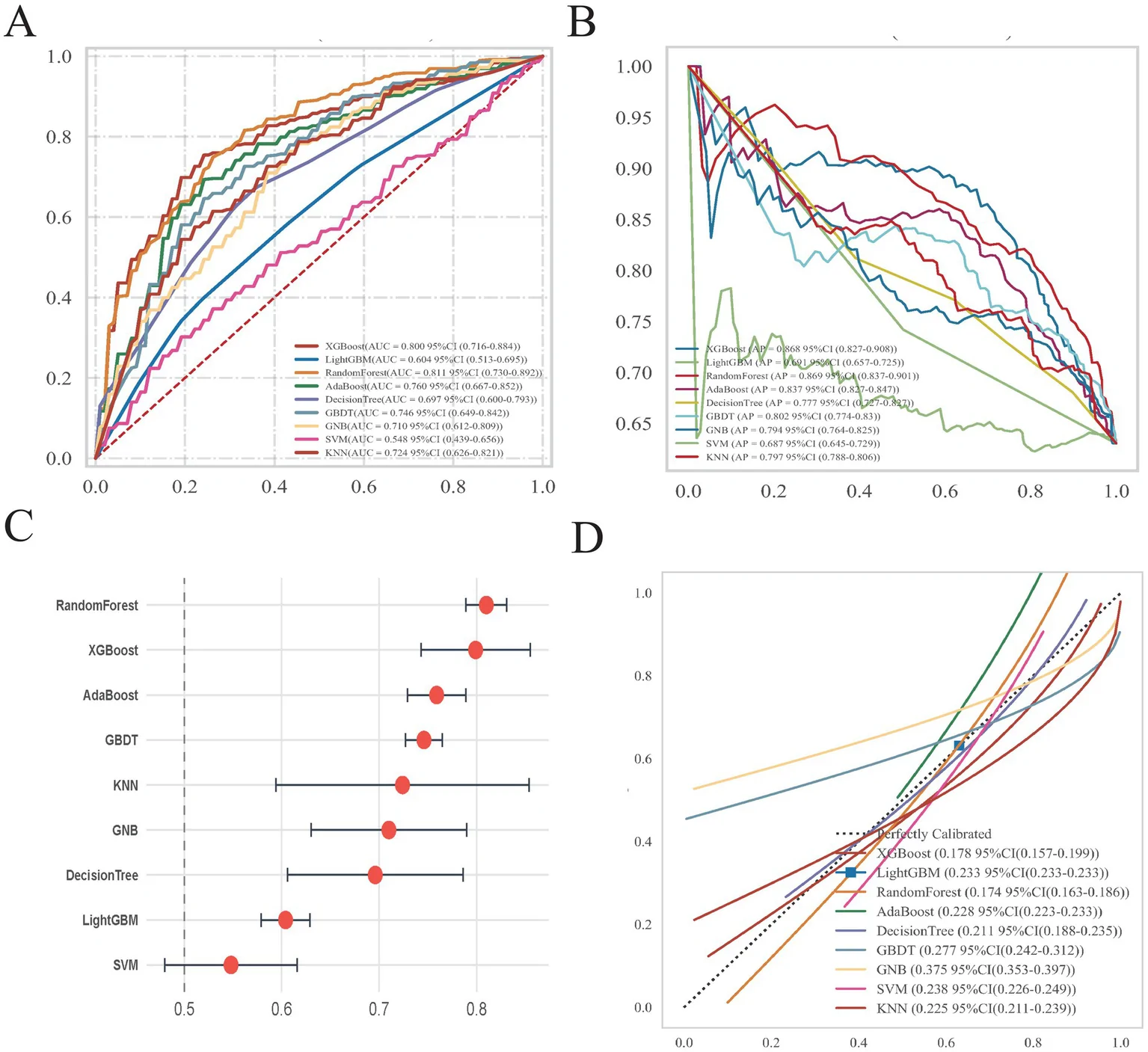
Performance evaluation of foundational machine learning models. **(A)** Receiver Operating Characteristic (ROC) curves and **(B)** Precision-Recall (PR) curves for the nine candidate base learners. **(C)** Forest plot summarizing AUC scores with 95% confidence intervals across all models. **(D)** Calibration curves evaluating the agreement between predicted SAP risks and observed outcomes.

DeLong test results (Supplementary Table S5) showed that while the RF model had the highest numerical AUC, its performance was not significantly different from other top-tier ensemble models such as XGBoost (*p* = 0.594) or AdaBoost (*p* = 0.293), as all pairwise *p*-values were well above the adjusted significance threshold of 0.0056.

### 3.4 Construction and performance

Given the statistical equivalence in independent predictive efficacy among the top-tier base learners, this study implemented a Blending Ensemble Strategy. We introduced Logistic Regression as the meta-learner to achieve the optimal calibration and fusion of predictions by learning the strategic weighting of these base models. The final Logistic-Blending model demonstrated outstanding discriminatory power in the validation cohort, achieving a superior AUC of 0.898 (95% CI: 0.845–0.951) (Figure 3A), which represented a substantial absolute improvement of 8.3% over the best single model (RF, AUC = 0.815) (Table 3). The multi-dimensional evaluation further confirmed the model’s robustness and clinical reliability. Calibration curves (Figure 3B) showed high consistency between predicted probabilities and observed SAP outcomes, with a Brier score of 0.214 (95% CI: 0.209–0.220). To assess clinical utility, Decision Curve Analysis (DCA) was performed (Figure 3C), and PR analysis (Figure 3D) yielded an AP of 0.777, indicating that the model maintains high precision even under potential class imbalance. Additionally, DCA demonstrated that the Blending model provides a superior net benefit across a wide range of threshold probabilities compared to treat-all or treat-none strategies. Furthermore, compared to the RF model, the Blending model achieved a significant enhancement in risk reclassification, with a categorical NRI of 0.165 (95% CI: 0.112–0.218, *p* < 0.001) and an IDI of 0.082 (95% CI: 0.054–0.110, *p* < 0.001). While maintaining a specificity of 0.851, the model achieved a sensitivity of 0.782 and an *F*1-score of 0.801. These results underscore the Blending strategy’s capacity to optimize decision boundaries and provide substantial incremental value for early SAP screening. In the sensitivity analysis evaluating the impact of data imputation, the model trained on the complete case analysis (CCA) dataset yielded performance comparable to the primary MissForest-imputed model. Specifically, in the internal test set, the CCA model achieved an AUC of 0.879 (95% CI: 0.816–0.942), which showed no statistically significant difference compared to the primary model (AUC = 0.898, DeLong test *p* = 0.284). Similar consistency was observed in the temporal validation cohort (*p* = 0.231), confirming that the imputation strategy did not introduce optimistic bias and demonstrated acceptable model stability (Supplementary Table S6).

**Figure 3:**
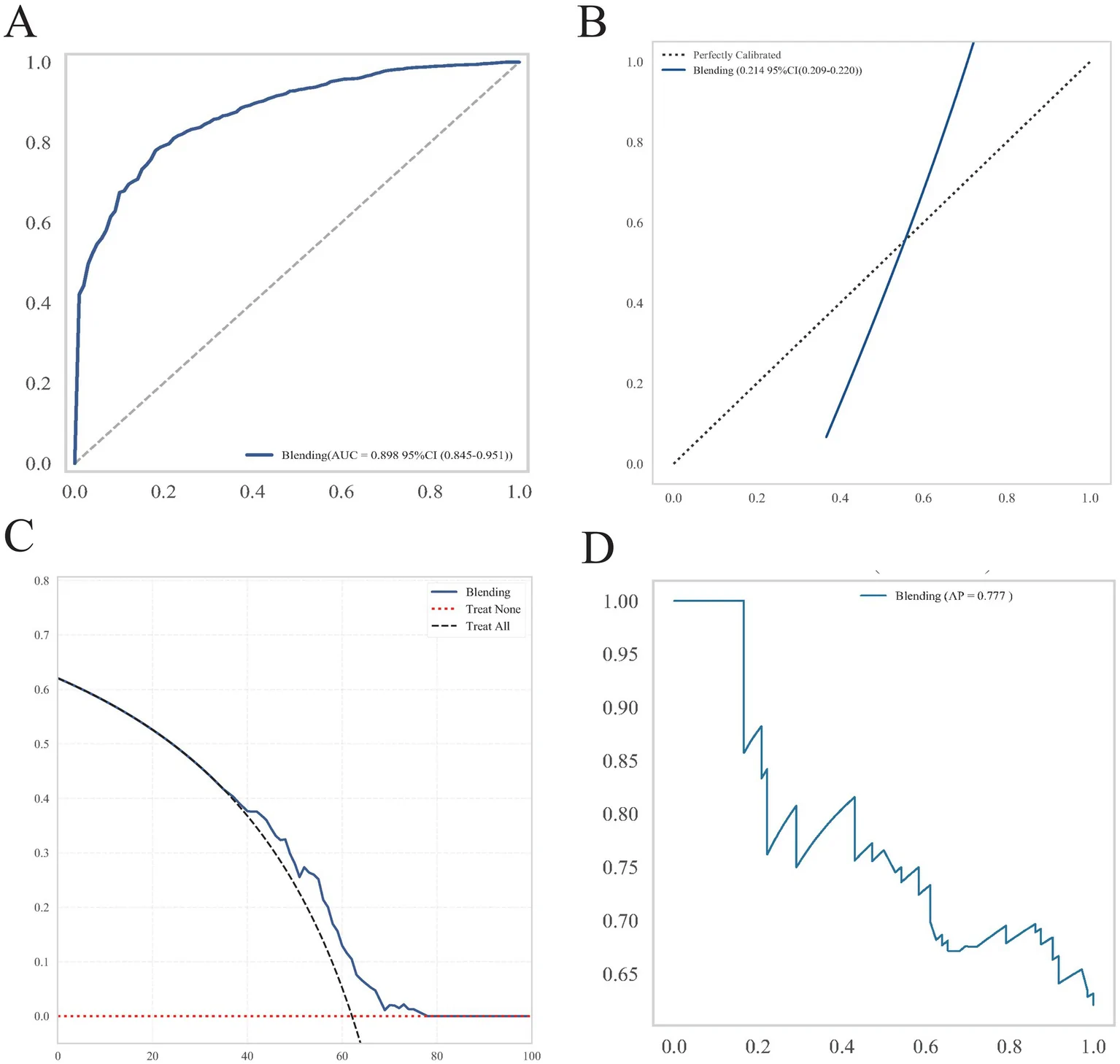
Predictive efficacy and clinical utility of the Blending Ensemble model. **(A)** ROC curve and **(B)** calibration curve demonstrating the discriminatory power and probabilistic alignment of the integrated model. **(C)** Decision Curve Analysis (DCA) illustrating clinical net benefit across various threshold probabilities. **(D)** Precision-Recall (PR) curve highlighting model performance under potential class imbalance.

**Table 3:** Comparative performance metrics between proposed blending ensemble model and individual base classifiers in validation cohort.

Model	AUC (95% CI)	Accuracy (95% CI)	Sensitivity (95% CI)	Specificity (95% CI)	*F*1 score	*p-*value
Blending ensemble	0.898 (0.845–0.951)	0.824 (0.785–0.863)	0.782 (0.735–0.829)	0.851 (0.806–0.896)	0.801	-
RF	0.815 (0.732–0.898)	0.712 (0.688–0.735)	0.662 (0.625–0.698)	0.795 (0.728–0.860)	0.742	0.012
XGBoost	0.804 (0.719–0.889)	0.748 (0.710–0.785)	0.746 (0.712–0.779)	0.749 (0.690–0.805)	0.789	< 0.001

AUC, area under the receiver operating characteristic curve; CI, confidence interval; RF, random forest; XGBoost, extreme gradient boosting.

### 3.5 Visual interpretation of key predictive

We utilized the SHAP framework to provide a multidimensional visualization of feature contributions (Figure 4), thereby elucidating the decision-making logic of the Blending Ensemble model. The SHAP beeswarm plot (Figure 4A) illustrates how feature magnitudes influence the predicted probability of SAP. Elevated levels (red) of hs-CRP, NIHSS, FIB, D-dimer, and SII were positively associated with higher SHAP values, indicating an increased risk of SAP. Similarly, advanced Age, Smoking history, and the presence of Dysphagia or COPD contributed to elevated risk scores. In contrast, higher ALB and MCHC levels were predominantly localized in the negative SHAP zone, suggesting a protective effect against SAP development. The SHAP interaction network (Figure 4B) reveals the complex interdependencies between key variables. The most prominent interaction was observed between hs-CRP and NIHSS, where the combination of systemic inflammation and neurological deficit severity synergistically amplified the risk of SAP. Furthermore, notable interaction strengths were identified between hs-CRP and both FIB and NLR, highlighting the interplay of various inflammatory pathways in the model’s decision boundary. The relative importance of clinical features was quantified using mean absolute SHAP values (Figures 4C,D). hs-CRP (17.6%), NIHSS (16.1%), and FIB (11.7%) emerged as the three primary drivers of the model’s predictions. These were followed by D-dimer (10.6%) and Age (7.3%), alongside inflammatory markers such as NLR and SII.

**Figure 4:**
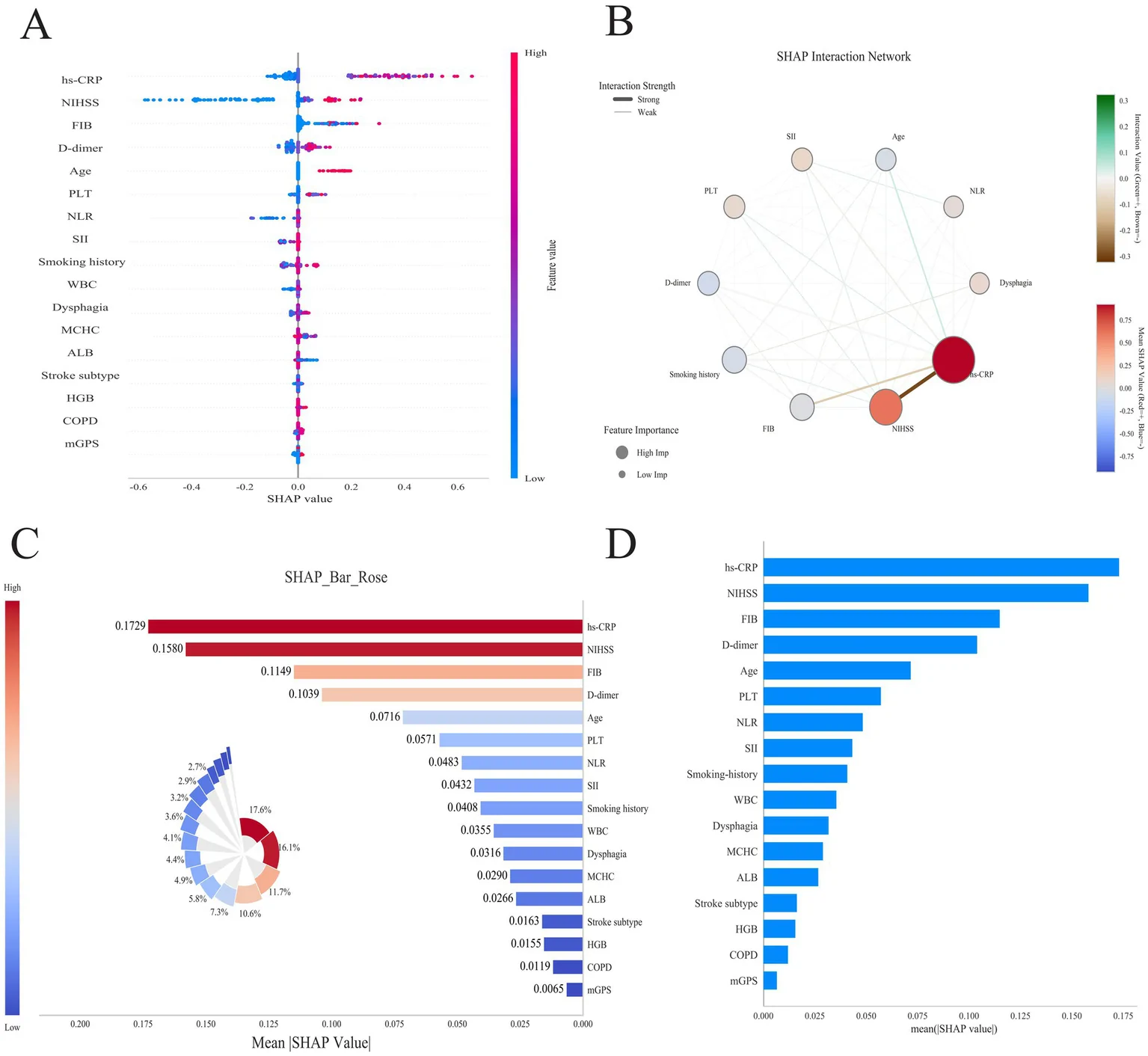
Multidimensional visualization of feature contributions via the SHAP framework. **(A)** SHAP beeswarm plot illustrating the directional effects and risk profiles of individual features. **(B)** SHAP interaction network revealing synergistic interdependencies between key variables. **(C,D)** Global feature importance ranking based on mean absolute SHAP values for the final Blending Ensemble model.

### 3.6 Evaluation of model generalizability

For further substantiation of the Blending ensemble model’s robustness in real-world clinical scenarios, we evaluated its performance across two distinct cohorts: an independent temporal validation cohort and an external multi-center validation cohort (Supplementary Table S7). A head-to-head comparison was conducted between the Blending Ensemble and the A2DS2 (implemented via logistic regression) in the temporal validation cohort (Table 4; Figure 5). The Blending model demonstrated a significant advantage in discriminatory performance. As illustrated in the ROC curves (Figure 5A), the Blending model achieved an AUC of 0.875 (95% CI: 0.841–0.910), significantly outperforming the A2DS2 (AUC = 0.805, 95% CI: 0.759–0.850). PR analysis (Figure 5B) further corroborated these findings, with the Blending model yielding a higher AP of 0.895 (95% CI: 0.880–0.901) compared to the A2DS2 (AP = 0.837, 95% CI: 0.826–0.848). To further quantify the incremental improvement over the traditional clinical scoring system, the NRI and IDI were calculated. Compared to the A2DS2, the Blending model achieved a significant enhancement in risk reclassification, with a categorical NRI of 0.142 (95% CI: 0.091–0.193, *p* < 0.001) and an IDI of 0.068 (95% CI: 0.042–0.094, *p* < 0.001). These metrics indicate that the Blending strategy more accurately reclassifies patients into their true risk categories, thereby reducing clinical misclassification. In terms of predictive accuracy, the Blending model maintained a superior balance between sensitivity and specificity. It attained a higher Accuracy (0.800) and a more robust *F*1-score (0.831). Notably, while the A2DS2 exhibited high sensitivity (0.803), its Specificity (0.699) was considerably lower than that of the Blending model (0.814). This suggests that the Blending model can more effectively minimize false positives while maintaining strong detection capabilities for SAP. Calibration curves (Figure 5C) demonstrated that while the A2DS2 showed a slightly lower Brier score of 0.187 (95% CI: 0.171–0.202) compared to the Blending model (0.214, 95% CI: 0.209–0.220), both models exhibited strong agreement between predicted probabilities and observed outcomes. Collectively, these results underscore that the Blending Ensemble strategy offers a more powerful and precise alternative to conventional clinical scores for SAP prediction.

**Table 4:** Predictive metrics of Blending ensemble model in prospective temporal.

Temporal validation cohort (*n* = 199)	AUC (95% CI)	Cutoff (95% CI)	Accuracy (95% CI)	Sensitivity (95% CI)	Specificity (95% CI)	PPV (95% CI)	NPV (95% CI)	*F*1 score (95% CI)	Kappa (95% CI)
A2DS2	0.805 (0.759–0.850)	0.563(0.533–0.592)	0.764(0.757–0.770)	0.803(0.766–0.841)	0.699(0.648–0.750)	0.814(0.794–0.833)	0.689(0.662–0.716)	0.808(0.797–0.818)	0.5(0.487–0.513)
Blending	0.875 (0.841–0.910)*	0.524(0.516-0.531)	0.8(0.791–0.810)	0.792(0.775–0.809)	0.814(0.794–0.834)	0.874(0.863–0.885)	0.707(0.692–0.722)	0.831(0.822–0.840)	0.589(0.569–0.608)

AUC, area under the receiver operating characteristic curve; CI, confidence interval; PPV, positive predictive value; NPV, negative predictive value. *p < 0.05.

**Figure 5:**
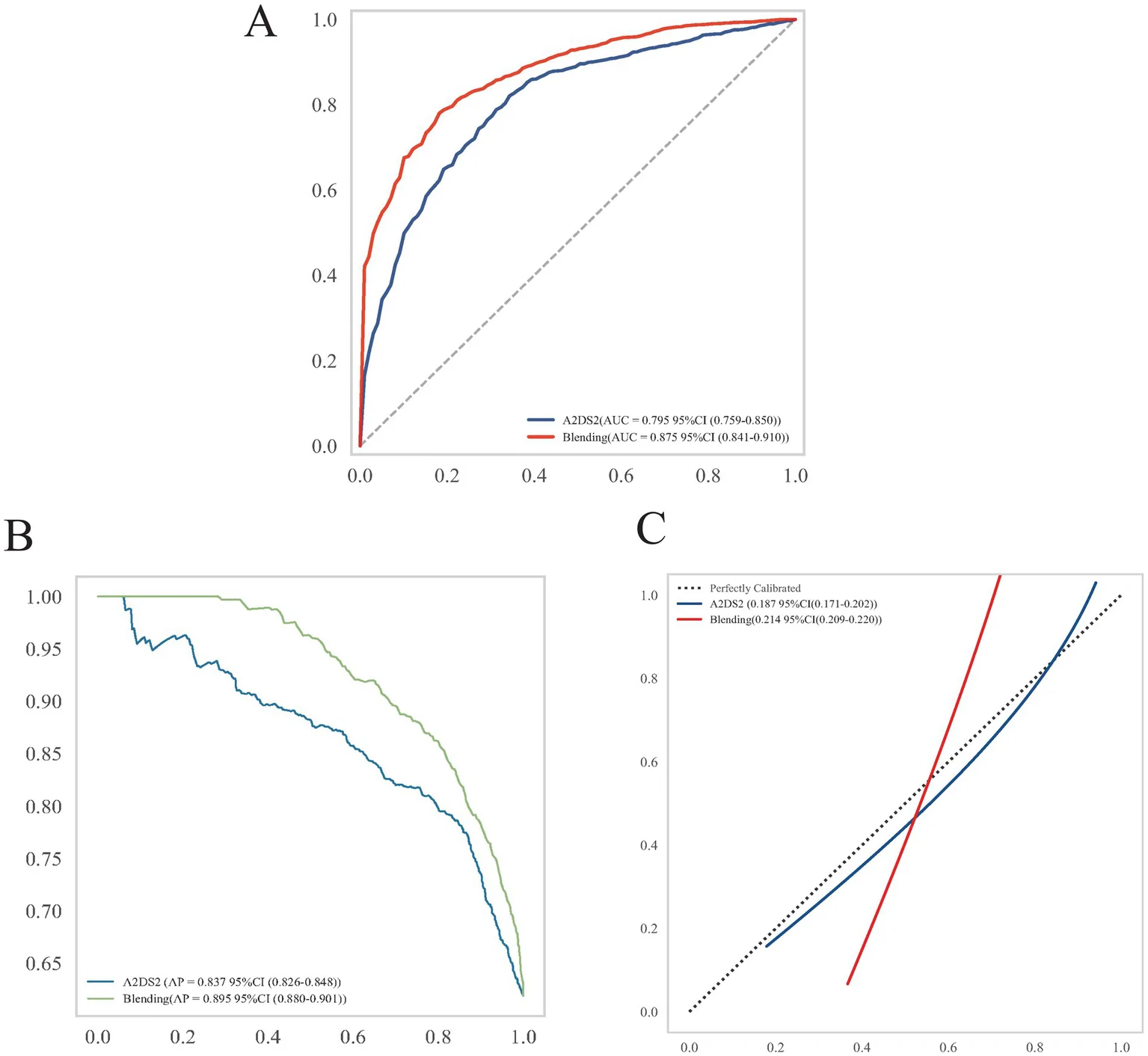
Head-to-head comparison between the blending ensemble model and the A2DS2. **(A)** ROC curves and **(B)** Precision-Recall (PR) curves comparing discriminatory performance in the temporal validation cohort. **(C)** Calibration curves evaluating the predictive consistency of both the ensemble model and the traditional clinical scoring system.

Generalizability was assessed by evaluating the Blending model in an independent external cohort (*n* = 287). The results demonstrated robust performance across different institutional data distributions (Table 5). The model maintained strong discriminatory power with an AUC of 0.825 (95% CI: 0.778–0.872) and an Accuracy of 0.767 (95% CI: 0.718–0.816). It exhibited balanced performance with a Sensitivity of 0.630 (95% CI: 0.535–0.725) and a Specificity of 0.840 (95% CI: 0.787–0.893). Notably, the high NPV of 0.809 (95% CI: 0.754–0.864) and an *F*1-score of 0.653 further confirm the model’s reliability for SAP screening in diverse clinical settings. These findings underscore the superior robustness and institutional generalizability of the proposed ensemble strategy. The confusion matrices are presented in Figure 6.

**Table 5:** Multi-center-based external validation cohort.

Performance metric	Point estimate	95% CI
AUC (95% CI)	0.825	(0.778–0.872)
Accuracy (95% CI)	0.767	(0.718–0.816)
Sensitivity (95% CI)	0.63	(0.535–0.725)
Specificity (95% CI)	0.84	(0.787–0.893)
PPV (95% CI)	0.677	(0.582–0.772)
NPV (95% CI)	0.809	(0.754–0.864)
*F*1 Score (95% CI)	0.653	(0.575–0.731)
Kappa (95% CI)	0.477	(0.375–0.579)

AUC, area under the receiver operating characteristic curve; CI, confidence interval; PPV, positive predictive value; NPV, negative predictive value.

**Figure 6:**
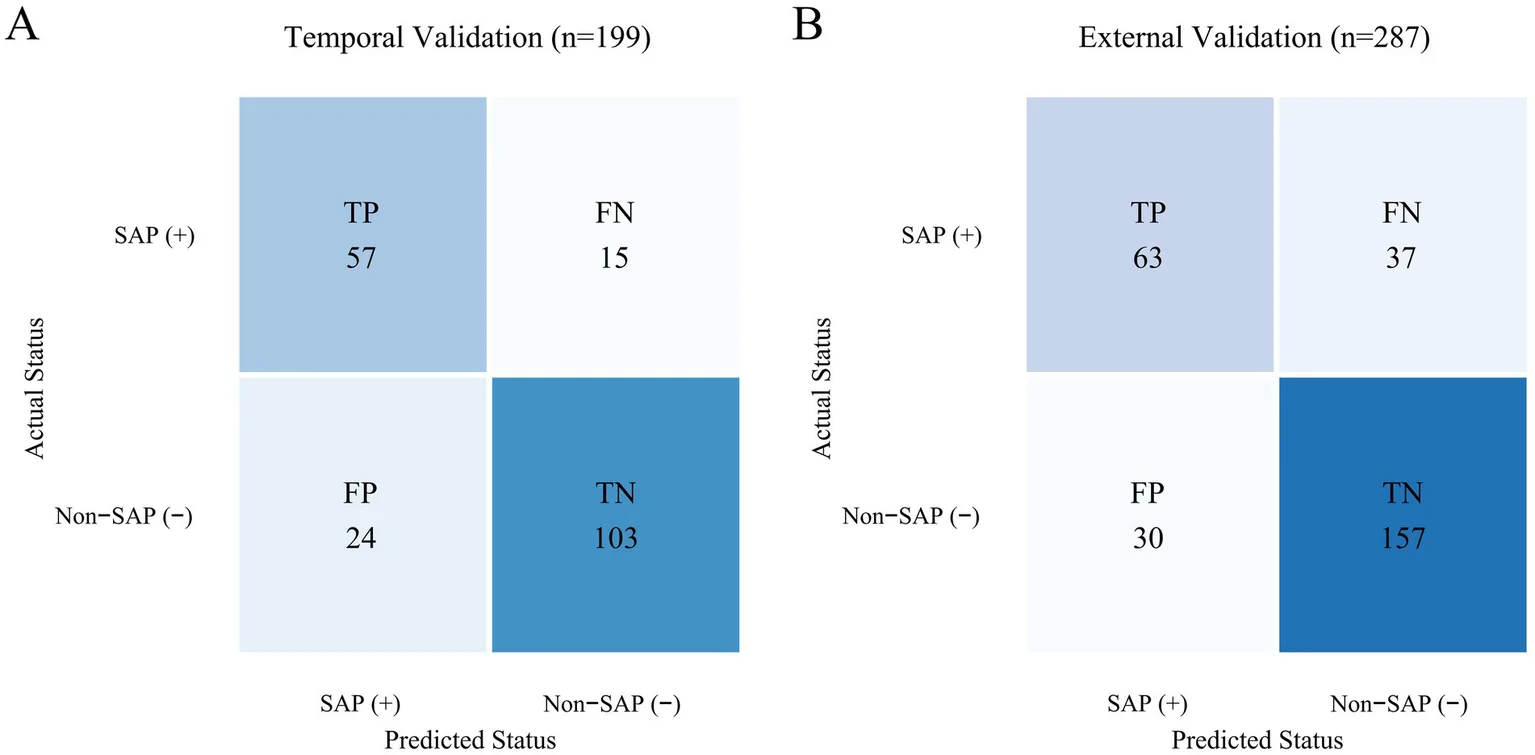
Confusion matrices across independent validation cohorts. **(A)** Classification results for the prospective temporal validation cohort (*n* = 199). **(B)** Classification results for the external multi-center validation cohort (*n* = 287), demonstrating the model’s geographical transportability.

### 3.7 Development and deployment of online prediction tool

To facilitate the clinical translation of our findings and bridge the gap between algorithmic complexity and daily practice, the optimal Blending Ensemble Model was deployed as an interactive, user-friendly web-based risk calculator.

Upon inputting the values of the 17 pivotal predictors, the backend system instantaneously computes the individual probability of developing SAP based on the ensemble algorithm. It subsequently performs automatic risk stratification, categorizing patients into “Low Risk” or “High Risk” groups based on the pre-established optimal threshold. Designed as an accessible Clinical Decision Support System, this tool empowers medical staff to rapidly identify high-risk individuals, thereby enabling the prompt initiation of preventive interventions. The tool is publicly available at: https://www.xsmartanalysis.com/predictive/preview/21.

## 4 Discussion

We developed and validated a SAP risk prediction model based on a Blending Ensemble Strategy, aiming to address the challenge of limited generalizability often encountered by single ML classifiers in complex clinical settings. Compared with traditional SAP risk scores, our model demonstrated significant performance improvements. Conventional scales rely heavily on static clinical features such as age and NIHSS, failing to adequately capture dynamic pathophysiological changes, which results in suboptimal sensitivity (14). In contrast, the Blending strategy adopted in this study synergizes the strengths of Bagging (RF) and Boosting (XGBoost) algorithms via linear weighted fusion. This approach effectively balances the Bias-Variance Tradeoff, thereby exhibiting superior robustness when handling high-dimensional clinical data.

Crucially, this study extended beyond internal testing to include both prospective temporal validation and rigorous external multi-center validation utilizing the Inspur Health Database. Results indicated that the model achieved an AUC of 0.875 and a remarkable sensitivity of 0.792 in the temporal validation set, significantly minimizing the risk of missed diagnosis. It is noteworthy that the external validation cohort exhibited distinct baseline characteristics compared to the development cohort, particularly significantly elevated NIHSS (*p* < 0.05). This divergence mirrors the heterogeneity often encountered in real-world clinical practice. However, despite such clinical variability, our model maintained a robust AUC of 0.825, avoiding the precipitous “performance decay” frequently reported in AI studies. Although the sensitivity in the external cohort showed marginal fluctuation, this is consistent with the inherent heterogeneity of real-world data and confirms that the proposed Blending ensemble strategy effectively mitigates the risk of overfitting while demonstrating robust generalizability.

While conventional research has primarily emphasized demographic characteristics and neurological deficits as the pathophysiological foundation of SAP, our SHAP global interpretability analysis reveals a more complex, multi-dimensional landscape. While Age has traditionally emerged as a predominant determinant in clinical scores like the A2DS2 (15), our model identifies systemic inflammatory and thrombotic markers, specifically hs-CRP, FIB, and D-dimer, as core drivers that carry higher predictive weight than age alone. Nevertheless, Age remains a critical factor that profoundly reflects the physiological state of “immunosenescence” (15). In elderly patients, the decline in alveolar macrophage phagocytosis combined with a diminished cough reflex creates a microenvironment highly susceptible to SAP (16). Our findings suggest that while these demographic factors are foundational, the integration of inflammatory and thrombotic profiles offers a more granular understanding of SAP pathogenesis. Concurrently, comorbidities such as COPD and a history of smoking imply a baseline impairment of the mucociliary clearance system, thereby predisposing patients to descending infections post-stroke (17–19).

Regarding stroke subtypes, our analysis revealed that Large Artery Atherosclerosis predominated in the SAP group, aligning with the epidemiological characteristic of a high burden of intracranial artery stenosis in the Chinese population (20). The massive cerebral infarction and severe hypoperfusion associated with this subtype often result in profound neurological impairment. Notably, the proportion of Small Artery Occlusion was also significant. This phenomenon can be elucidated by the impact of infarcts in critical functional areas: despite a small total volume, damage to pivotal regions can interrupt the corticobulbar tract, resulting in severe pseudobulbar palsy and dysphagia, thereby dramatically amplifying pneumonia risk (21, 22).

The high predictive weights assigned to NIHSS and dysphagia directly quantify the severity of central nervous system injury and its clinical sequelae (23). Severe neurological damage contributes to SAP through a dual mechanism: physically, it induces bulbar paralysis and subsequent swallowing dysfunction; systemically, it activates the “Brain-Lung Axis.” The SHAP interaction network (Figure 4B) specifically identified the strongest synergistic effect between hs-CRP and NIHSS, suggesting that sympathetic overactivation triggers local immune dysregulation, creating a permissive environment for bacterial colonization (24, 25).

Furthermore, SHAP analysis elucidated the profound immuno-thrombotic pathological mechanisms underlying SAP. Our results identified hs-CRP, SII, and NLR as heavily weighted predictors (26, 27). Following an acute stroke, the neuroendocrine storm induces apoptosis and functional exhaustion of peripheral lymphocytes while priming neutrophils to release Neutrophil Extracellular Traps (28). As composite indices, SII and NLR simultaneously capture this “pro-inflammatory/immuno-suppressive” disequilibrium, offering superior predictive value compared to isolated leukocyte counts (29). This state of immunoparalysis disrupts systemic homeostasis and compromises pulmonary defense barriers (8).

Intricately linked to this inflammatory cascade is “Immunothrombosis,” which explains the high ranking of FIB and D-dimer in our model (30). These markers are integral players in the disease process (31). The hyperinflammatory state activates the coagulation cascade, precipitating a rise in D-dimer levels (32). As a marker for hypercoagulability, elevated D-dimer indicates ongoing thrombosis within the microvasculature (33). This microcirculatory impairment induces pulmonary hypoperfusion and compromises local immune cell recruitment, rendering the lung a highly susceptible “target organ” for infection.

SHAP analysis further illuminated the predictive value of hematological indices, corroborating that reduced HGB and abnormal MCHC are risk factors for SAP. The underlying mechanism may be linked to “Hypoxia-Susceptibility Coupling,” where a decline in HGB induces tissue hypoxia, suppressing the bactericidal functions of alveolar macrophages (34). Moreover, reduced MCHC may suggest a chronic depletion state, further compromising the host’s anti-infection defense (35).

Beyond these indices, serum ALB exhibited a significant inverse correlation with SAP risk. Hypoalbuminemia serves as a surrogate for malnutrition and implies a scarcity of substrates for immunoglobulin synthesis (36–38). Our study observed that the mGPS, combining CRP and ALB, was elevated in the SAP group, underscoring that the post-stroke hypermetabolic state accelerates protein catabolism (39). Mechanistically, hypoalbuminemia may facilitate fluid exudation into the alveoli by diminishing plasma colloid osmotic pressure (40). Consequently, the coexistence of hypoalbuminemia and elevated inflammatory markers signals a critical state of “defense system decompensation” (41).

In conclusion, the integrated model constructed in this study synergizes novel composite inflammatory indices, coagulation parameters, anemia-related markers, and traditional neurological scores, thereby significantly enhancing the efficacy of early SAP recognition. To facilitate its clinical translation, we have developed an online visual risk assessment tool. Clinicians need only input the patient’s key metrics for the system to perform real-time risk stratification. For individuals stratified as “high-risk,” the clinical team can preemptively initiate targeted interventions. These include intensified airway management and precision nutritional support, thereby offering the potential to intercept infection and improve patient prognosis.

Several limitations of this study merit objective acknowledgment. First, given the retrospective nature of the design, although we implemented consecutive enrollment and prospective temporal validation to mitigate biases, the selection bias and information bias inherent to retrospective data collection cannot be entirely eliminated. Second, to prioritize data quality, the external validation relied on a stratified random sampling strategy rather than a full-scale dataset analysis. While this sample maintains statistical representativeness, it may inherently restrict the statistical power for certain granular subgroup analyses. Third, the inability to prospectively unify swallowing assessment across external cohorts may have introduced inter-observer and inter-center heterogeneity, but its impact was attenuated by using a binary variable for dysphagia. Future initiatives will focus on conducting large-scale, multi-center prospective studies to further calibrate and refine the model’s generalizability.

## 5 Conclusion

In summary, this study successfully developed and validated a high-performance SAP prediction model leveraging Blending Ensemble Learning. By synergizing novel composite inflammatory markers, coagulation parameters, anemia-related indices, and traditional neurological scores, the model demonstrated superior predictive efficacy, robust generalizability, and clear biological interpretability across multi-center cohorts. To dismantle technical barriers and facilitate clinical translation, we have deployed the model as an online visual risk assessment tool. This instrument enables clinicians to perform real-time risk stratification by simply inputting key metrics. Clinically, this tool serves as an objective guide for early identification of high-risk individuals, guiding preemptive strategies such as strengthened airway care and precise nutritional support to block infection and optimize prognoses.

## Data Availability

The raw data supporting the conclusions of this article will be made available by the authors, without undue reservation.
