# Supplementary material for: Construction and multi-center validation of a model based on blending ensemble learning for predicting stroke-associated pneumonia following acute ischemic stroke
Source: Front Neurol. 2026 Sep 18;17:1841730. doi: 10.3389/fneur.2026.1841730 (PMC13630648; doi:10.3389/fneur.2026.1841730)
Supplement: Supplementary file 1 [file Table_1.DOCX]

**Construction and Multi-center Validation of a Model Based on Blending Ensemble Learning for Predicting Stroke-Associated Pneumonia Following Acute Ischemic Stroke**

**Multimedia Appendix 1**

**Contents**

[Supplemental Table S1. Software specifications of the machine learning models.	1](#_Toc6250)

[Supplemental Table S2. Comprehensive Baseline Characteristics.	2](#_Toc27303)

[Supplemental Table S3. Stability of coefficients for selected predictors of stroke-associated pneumonia across stratified 5-fold cross-validation.	6](#_Toc9398)

[Supplemental Table S4. Multicollinearity Diagnostic for Candidate Predictors Based on Variance Inflation Factor (VIF).	8](#_Toc20401)

[Supplemental Table S5. Pairwise comparisons of the AUC between nine machine learning models using the DeLong test.	9](#_Toc18525)

[Supplemental Table S6. Sensitivity Analysis of Missing Data Handling.	10](#_Toc11812)

[Supplemental Table S7. Baseline Characteristics and Predictors Across Cohorts.	11](#_Toc1740)

# Supplemental Table S1. Software specifications of the machine learning models.

| Model | Library | Version | Hyperparameters |
| --- | --- | --- | --- |
| Blending Ensemble | scikit-learn | 1.1.3 | C = 10.0, l1_ratio = None, max_iter = 50, penalty = ‘l2’, solver = ‘lbfgs’, tol = 1e-06 |
| RF | scikit-learn | Latest (v1.6.0) | Criterion = ‘gini’, n_estimators = 100, max_depth = None, max_features = ‘sqrt’, min_samples_split = 2 |
| Gradient Boosting | scikit-learn | Latest (v1.6.0) | learning_rate = 2, n_estimators = 100, max_depth = 1, loss = ‘exponential’ |
| KNN | scikit-learn | Latest (v1.6.0) | n_neighbors = 4, weights = ‘distance’, algorithm = ‘auto’, p = 2 |
| XGBoost | xgboost | Latest (v2.1.0) | learning_rate = 0.3, n_estimators = 20, max_depth = 4, subsample = 1, colsample_bytree = 1 |
| AdaBoost | scikit-learn | Latest (v1.6.0) | learning_rate = 0.3, n_estimators = 50 |
| DT | scikit-learn | Latest (v1.6.0) | criterion = ‘gini’, max_depth = 20, min_samples_split = 50 |
| SVM | scikit-learn | Latest (v1.6.0) | C = 1.0, kernel = ‘rbf’, gamma = ‘scale’, probability = True |
| GNB | scikit-learn | Latest (v1.6.0) | var_smoothing = 1e-07 |
| LightGBM | lightgbm | Latest (v4.6.0) | boosting_type = ‘gbdt’, learning_rate = 2, n_estimators = 5, num_leaves = 5 |

Note: AdaBoost: Adaptive Boosting; DT: Decision Tree; RF: Random Forest; GNB: Gaussian Naive Bayes; KNN: K-Nearest Neighbors; XGBoost: Extreme Gradient Boosting; LightGBM: Light Gradient Boosting Machine; SVM: Support Vector Machine.

Model Configuration: The Blending ensemble model employed Logistic Regression as the meta-learner, configured with L_2_ regularization (*C* = 10.0) and the lbfgs solver.

Hyperparameters: Key parameters for each model are listed; parameters not specified were set to their default values.

# Supplemental Table S2. Comprehensive Baseline Characteristics.

| Characteristics | Total Cohort  (n=1,123) | SAP Group  (n=236) | Non-SAP Group  (n=887) | Missing, Total / SAP / Non-SAP (n,%) | *p* value |
| --- | --- | --- | --- | --- | --- |
| Demographics |  |  |  |  |  |
| Age, years, median (IQR) | 69 (61–77) | 71 (62–81) | 68 (61–75) | 0 (0.00%) / 0 (0.00%) / 0 (0.00%) | 0.002 |
| Sex , n (%) (Male) | 714 (63.58%) | 166 (70.34%) | 549 (61.89%) | 0 (0.00%) / 0 (0.00%) / 0 (0.00%) | 0.020 |
| Marital Status, n (%) |  |  |  | 0 (0.00%) / 0 (0.00%) / 0 (0.00%) | 0.652 |
| Married | 902 (80.32%) | 188 (79.66%) | 714 (80.50%) |  |  |
| Single | 200 (17.80%) | 45 (19.07%) | 155 (17.47%) |  |  |
| Widowed | 21 (1.87%) | 3 (1.27%) | 18 (2.03%) |  |  |
| Smoking History, n (%) | 378 (33.66%) | 36 (15.25%) | 342 (38.56%) | 12 (1.07%) / 2 (0.85%) / 10 (1.13%) | <0.001 |
| Alcohol Consumption, n (%) | 285 (25.38%) | 33 (13.98%) | 252 (28.41%) | 12 (1.07%) / 2 (0.85%) / 10 (1.13%) | <0.001 |
| Comorbidities |  |  |  |  |  |
| HTN, n (%) | 920 (81.92%) | 210 (88.98%) | 710 (80.05%) | 0 (0.00%) / 0 (0.00%) / 0 (0.00%) | 0.007 |
| DM, n (%) | 451 (40.16%) | 118 (50.00%) | 333 (37.54%) | 0 (0.00%) / 0 (0.00%) / 0 (0.00%) | 0.003 |
| HLD, n (%) | 127 (11.31%) | 38 (16.10%) | 89 (10.03%) | 0 (0.00%) / 0 (0.00%) / 0 (0.00%) | 0.011 |
| CHD, n (%) | 294 (26.18%) | 57 (24.15%) | 237 (26.72%) | 0 (0.00%) / 0 (0.00%) / 0 (0.00%) | 0.420 |
| AF, n (%) | 50 (4.45%) | 20 (8.47%) | 30 (3.38%) | 0 (0.00%) / 0 (0.00%) / 0 (0.00%) | 0.001 |
| HF, n (%) | 31 (2.76%) | 3 (1.27%) | 28 (3.16%) | 0 (0.00%) / 0 (0.00%) / 0 (0.00%) | 0.184 |
| MI, n (%) | 20 (1.78%) | 2 (0.85%) | 18 (2.03%) | 0 (0.00%) / 0 (0.00%) / 0 (0.00%) | 0.354 |
| Prior Stroke/Hemorrhage, n (%) | 540 (48.09%) | 147 (62.29%) | 393 (44.30%) | 0 (0.00%) / 0 (0.00%) / 0 (0.00%) | <0.001 |
| COPD, n (%) | 73 (6.50%) | 26 (11.02%) | 47 (5.30%) | 0 (0.00%) / 0 (0.00%) / 0 (0.00%) | 0.002 |
| Clinical Features |  |  |  |  |  |
| NIHSS, median (IQR) | 3 (1–6) | 7 (4–9) | 2 (1–3.5) | 0 (0.00%) / 0 (0.00%) / 0 (0.00%) | <0.001 |
| A2DS2, median (IQR) | 2 (1–4) | 4 (2–5) | 1 (1–2) | 0 (0.00%) / 0 (0.00%) / 0 (0.00%) | <0.001 |
| Dysphagia, n (%) | 110 (9.80%) | 44 (18.64%) | 66 (7.44%) | 0 (0.00%) / 0 (0.00%) / 0 (0.00%) | <0.001 |
| Stroke Subtype (TOAST), n (%) |  |  |  | 0 (0.00%) / 0 (0.00%) / 0 (0.00%) | 0.021 |
| CE | 158 (14.07%) | 34 (14.40%) | 124 (13.98%) |  |  |
| SAO | 179 (15.94%) | 45 (19.07%) | 134 (15.11%) |  |  |
| SUE | 37 (3.29%) | 2 (0.85%) | 35 (3.95%) |  |  |
| LAA | 733 (65.27%) | 155 (65.68%) | 578 (65.16%) |  |  |
| SOE | 16 (1.42%) | 0 (0.00%) | 16 (1.80%) |  |  |
| mGPS, n (%) |  |  |  | 31 (2.76%) / 6 (2.54%) / 25 (2.82%) | <0.001 |
| 0 | 1030 (91.72%) | 175 (74.15%) | 855 (96.39%) |  |  |
| 1 | 60 (5.34%) | 36 (15.25%) | 24 (2.71%) |  |  |
| 2 | 33 (2.94%) | 25 (10.60%) | 8 (0.90%) |  |  |
| Hematology |  |  |  |  |  |
| WBC, ×10⁹/L, median (IQR) | 6.81 (5.73–8.18) | 6.98 (5.88–8.62) | 6.73 (5.66–8.02) | 16 (1.42%) / 3 (1.27%) / 13 (1.47%) | 0.035 |
| RBC, ×10¹²/L, median (IQR) | 4.48 (4.09–4.84) | 4.35 (3.93–4.75) | 4.52 (4.18–4.89) | 16 (1.42%) / 3 (1.27%) / 13 (1.47%) | <0.001 |
| HGB, g/L, median (IQR) | 135 (124–146) | 129 (116–142) | 137 (126–148) | 16 (1.42%) / 3 (1.27%) / 13 (1.47%) | <0.001 |
| HCT, %, median (IQR) | 40.8 (37.2–43.5) | 38.9 (34.9–42.2) | 41.4 (38.3–44.0) | 16 (1.42%) / 3 (1.27%) / 13 (1.47%) | <0.001 |
| MCV, fL, median (IQR) | 90.5 (87.5–93.9) | 90.0 (87.0–93.1) | 90.7 (87.8–94.3) | 16 (1.42%) / 3 (1.27%) / 13 (1.47%) | 0.038 |
| MCH, pg, median (IQR) | 30.2 (29.2–31.2) | 30.1 (29.0–31.1) | 30.3 (29.4–31.4) | 16 (1.42%) / 3 (1.27%) / 13 (1.47%) | 0.010 |
| MCHC, g/L, median (IQR) | 326 (315–338) | 332 (325–340) | 318 (310–324) | 16 (1.42%) / 3 (1.27%) / 13 (1.47%) | 0.003 |
| PLT, ×10⁹/L, median (IQR) | 215 (178–253) | 223 (188–265) | 208.5 (177–246) | 16 (1.42%) / 3 (1.27%) / 13 (1.47%) | 0.001 |
| RDW-SD, fL, median (IQR) | 43.0 (40.6–45.4) | 43.6 (41.1–46.3) | 42.7 (40.4–45.1) | 18 (1.60%) / 4 (1.69%) / 14 (1.58%) | 0.010 |
| RDW-CV, %, median (IQR) | 12.9 (12.4–13.6) | 13.2 (12.6–14.0) | 12.8 (12.3–13.3) | 18 (1.60%) / 4 (1.69%) / 14 (1.58%) | <0.001 |
| PDW, fL, median (IQR) | 11.5 (10.3–12.8) | 11.5 (10.1–12.9) | 11.5 (10.4–12.8) | 20 (1.78%) / 4 (1.69%) / 16 (1.80%) | 0.721 |
| MPV, fL, median (IQR) | 10.1 (9.6–10.7) | 10.1 (9.5–10.8) | 10.1 (9.6–10.7) | 20 (1.78%) / 4 (1.69%) / 16 (1.80%) | 0.942 |
| P-LCR, %, median (IQR) | 25.7 (21.4–30.7) | 25.2 (20.8–31.4) | 25.8 (21.6–30.4) | 22 (1.96%) / 5 (2.12%) / 17 (1.92%) | 0.769 |
| PCT, %, median (IQR) | 0.22 (0.18–0.25) | 0.22 (0.19–0.27) | 0.21 (0.18–0.24) | 20 (1.78%) / 4 (1.69%) / 16 (1.80%) | 0.001 |
| NEUT, ×10⁹/L, median (IQR) | 4.28 (3.45–5.46) | 4.58 (3.69–5.94) | 4.12 (3.28–5.33) | 16 (1.42%) / 3 (1.27%) / 13 (1.47%) | 0.001 |
| LYMPH, ×10⁹/L, median (IQR) | 1.65 (1.26–2.13) | 1.49 (1.15–1.94) | 1.74 (1.36–2.24) | 16 (1.42%) / 3 (1.27%) / 13 (1.47%) | <0.001 |
| MONO, ×10⁹/L, median (IQR) | 0.50 (0.39–0.64) | 0.53 (0.40–0.70) | 0.48 (0.38–0.62) | 16 (1.42%) / 3 (1.27%) / 13 (1.47%) | 0.004 |
| EO, ×10⁹/L, median (IQR) | 0.12 (0.06–0.20) | 0.13 (0.07–0.22) | 0.11 (0.06–0.18) | 16 (1.42%) / 3 (1.27%) / 13 (1.47%) | 0.134 |
| BASO, ×10⁹/L, median (IQR) | 0.03 (0.02–0.04) | 0.03 (0.02–0.04) | 0.03 (0.02–0.04) | 16 (1.42%) / 3 (1.27%) / 13 (1.47%) | 0.036 |
| MNEUT, ×10⁹/L, median (IQR) | 4.10 (3.21–5.20) | 4.10 (3.26–5.40) | 4.10 (3.21–5.17) | 18 (1.60%) / 4 (1.69%) / 14 (1.58%) | 0.587 |
| NEUT, %, median (IQR) | 64.4 (56.8–71.1) | 66.9 (60.3–75.4) | 63.1 (55.5–69.1) | 16 (1.42%) / 3 (1.27%) / 13 (1.47%) | <0.001 |
| LYMPH, %, median (IQR) | 25.4 (19.1–31.7) | 22.5 (15.9–29.1) | 27.0 (21.2–32.8) | 16 (1.42%) / 3 (1.27%) / 13 (1.47%) | <0.001 |
| MONO, %, median (IQR) | 7.40 (6.03–8.88) | 7.55 (6.10–9.20) | 7.30 (6.00–8.70) | 16 (1.42%) / 3 (1.27%) / 13 (1.47%) | 0.213 |
| Biochemistry |  |  |  |  |  |
| K, mmol/L, median (IQR) | 3.82 (3.58–4.05) | 3.86 (3.59–4.08) | 3.81 (3.57–4.03) | 25 (2.23%) / 5 (2.12%) / 20 (2.25%) | 0.142 |
| Na, mmol/L, median (IQR) | 140 (139–142) | 140 (138–142) | 140 (139–142) | 25 (2.23%) / 5 (2.12%) / 20 (2.25%) | 0.771 |
| CO2CP, mmol/L, median (IQR) | 21.0 (19.5–22.6) | 21.0 (19.7–22.9) | 20.9 (19.5–22.4) | 28 (2.49%) / 6 (2.54%) / 22 (2.48%) | 0.210 |
| GLU, mmol/L, median (IQR) | 6.23 (5.38–8.63) | 6.18 (5.29–9.70) | 6.24 (5.39–8.39) | 22 (1.96%) / 4 (1.69%) / 18 (2.03%) | 0.997 |
| Urea, mmol/L, median (IQR) | 5.18 (4.22–6.36) | 5.50 (4.14–7.17) | 5.08 (4.27–6.19) | 25 (2.23%) / 5 (2.12%) / 20 (2.25%) | 0.126 |
| CR, µmol/L, median (IQR) | 72.8 (59.7–88.7) | 72.8 (60.5–96.2) | 72.6 (59.2–87.1) | 25 (2.23%) / 5 (2.12%) / 20 (2.25%) | 0.332 |
| TP, g/L, median (IQR) | 65.0 (60.7–69.5) | 64.0 (60.1–68.3) | 65.5 (61.2–69.7) | 27 (2.40%) / 6 (2.54%) / 21 (2.37%) | 0.016 |
| ALB, g/L, median (IQR) | 39.0 (36.2–41.6) | 37.5 (33.2–40.6) | 39.6 (37.2–42.2) | 27 (2.40%) / 6 (2.54%) / 21 (2.37%) | <0.001 |
| CHOL, mmol/L, median (IQR) | 4.36 (3.52–5.14) | 4.35 (3.54–5.22) | 4.38 (3.51–5.08) | 32 (2.85%) / 7 (2.97%) / 25 (2.82%) | 0.569 |
| TG, mmol/L, median (IQR) | 1.42 (1.05–1.82) | 1.43 (1.07–1.85) | 1.40 (1.05–1.80) | 32 (2.85%) / 7 (2.97%) / 25 (2.82%) | 0.522 |
| HDL-C, mmol/L, median (IQR) | 1.07 (0.89–1.25) | 1.04 (0.85–1.24) | 1.08 (0.92–1.25) | 32 (2.85%) / 7 (2.97%) / 25 (2.82%) | 0.109 |
| LDL-C, mmol/L, median (IQR) | 2.63 (2.05–3.27) | 2.57 (2.09–3.25) | 2.66 (1.99–3.29) | 32 (2.85%) / 7 (2.97%) / 25 (2.82%) | 0.677 |
| GLB, g/L, median (IQR) | 26.5 (23.4–29.8) | 27.1 (24.4–30.7) | 26.1 (23.0–29.2) | 27 (2.40%) / 6 (2.54%) / 21 (2.37%) | 0.002 |
| A/G, median (IQR) | 1.48 (1.30–1.69) | 1.37 (1.18–1.60) | 1.54 (1.35–1.73) | 27 (2.40%) / 6 (2.54%) / 21 (2.37%) | <0.001 |
| AG, mmol/L, median (IQR) | 12.0 (10.7–13.4) | 12.2 (10.9–13.6) | 12.0 (10.5–13.3) | 28 (2.49%) / 6 (2.54%) / 22 (2.48%) | 0.087 |
| OSM, mOsm/L, median (IQR) | 292 (289–296) | 292 (289–296) | 292 (289–296) | 28 (2.49%) / 6 (2.54%) / 22 (2.48%) | 0.678 |
| eGFR, mL/min/1.73m², median (IQR) | 89 (79–97) | 85 (79–94) | 90 (79–97) | 25 (2.23%) / 5 (2.12%) / 20 (2.25%) | 0.119 |
| Coagulation |  |  |  |  |  |
| PT, s, median (IQR) | 11.2 (10.7–12.0) | 11.3 (10.6–12.2) | 11.2 (10.8–11.9) | 42 (3.74%) / 9 (3.81%) / 33 (3.72%) | 0.642 |
| APTT, s, median (IQR) | 26.8 (23.2–30.9) | 26.7 (21.8–31.6) | 26.9 (23.7–30.8) | 42 (3.74%) / 9 (3.81%) / 33 (3.72%) | 0.180 |
| TT, s, median (IQR) | 18.4 (17.5–19.3) | 18.1 (17.2–19.2) | 18.6 (17.7–19.3) | 42 (3.74%) / 9 (3.81%) / 33 (3.72%) | 0.004 |
| FIB, g/L, median (IQR) | 2.83 (2.38–3.35) | 3.18 (2.74–3.89) | 2.62 (2.34–3.10) | 42 (3.74%) / 9 (3.81%) / 33 (3.72%) | <0.001 |
| PT, %, median (IQR) | 97.70 (90.10–104.50) | 97.90 (90.10–104.80) | 97.70 (90.30–104.10) | 42 (3.74%) / 9 (3.81%) / 33 (3.72%) | 0.991 |
| D-dimer, mg/L, median (IQR) | 0.45 (0.24–1.41) | 0.76 (0.34–3.03) | 0.35 (0.20–0.94) | 58 (5.16%) / 14 (5.93%) / 44 (4.96%) | <0.001 |
| Inflammation & Novel Indices |  |  |  |  |  |
| CRP, mg/L, median (IQR) | 10 (10–10) | 10 (10–18.3) | 10 (10–10) | 65 (5.79%) / 15 (6.36%) / 50 (5.64%) | <0.001 |
| hs-CRP, mg/L, median (IQR) | 0.69 (0.50–3.40) | 4.23 (1.69–9.12) | 0.50 (0.50–0.55) | 72 (6.41%) / 17 (7.20%) / 55 (6.20%) | <0.001 |
| NLR, median (IQR) | 2.56 (1.80–3.64) | 3.04 (2.02–4.62) | 2.37 (1.70–3.20) | 16 (1.42%) / 3 (1.27%) / 13 (1.47%) | <0.001 |
| PLR, median (IQR) | 127 (99–168) | 147 (110–197) | 119 (91–153) | 16 (1.42%) / 3 (1.27%) / 13 (1.47%) | <0.001 |
| MLR, median (IQR) | 0.29 (0.22–0.41) | 0.35 (0.24–0.51) | 0.27 (0.22–0.36) | 16 (1.42%) / 3 (1.27%) / 13 (1.47%) | <0.001 |
| SIRI, median (IQR) | 1.25 (0.81–1.99) | 1.51 (0.94–3.05) | 1.13 (0.77–1.66) | 16 (1.42%) / 3 (1.27%) / 13 (1.47%) | <0.001 |
| SII, median (IQR) | 545 (365–794) | 681 (421–1021) | 474 (341–704) | 16 (1.42%) / 3 (1.27%) / 13 (1.47%) | <0.001 |

# Note: SAP: stroke-associated pneumonia; IQR: interquartile range; COPD: chronic obstructive pulmonary disease; HTN: Hypertension; DM: Diabetes Mellitus; HLD: Hyperlipidemia; CHD: Coronary Heart Disease; AF: Atrial Fibrillation; HF: heart failure; MI: Myocardial Infarction; NIHSS: National Institutes of Health Stroke Scale; A2DS2: Age: Atrial fibrillation: Dysphagia: Sex: Stroke Severity; TOAST: Trial of Org 10172 in Acute Stroke Treatment; mGPS: modified Glasgow Prognostic Score; WBC: white blood cell; RBC: red blood cell; HGB: Hemoglobin; HCT: Hematocrit; MCV: mean corpuscular volume; MCH: mean corpuscular hemoglobin; MCHC: mean corpuscular hemoglobin concentration; PLT: Platelets; RDW-SD: red blood cell distribution width-standard deviation; RDW-CV: red blood cell distribution width-coefficient of variation; PDW: platelet distribution width; MPV: mean platelet volume; P-LCR: platelet-large cell ratio; PCT: plateletcrit; NEUT: neutrophil count; LYMPH: lymphocyte count; MONO: monocyte count; EO: eosinophil count; BASO: basophil count; MNEUT: mature neutrophil count; NEUT%: neutrophil percentage; LYMPH%: lymphocyte percentage; MONO%: monocyte percentage; K: potassium; Na: sodium; CO2CP: carbon dioxide combining power; GLU: glucose; Urea: urea; CR: creatinine; TP: total protein; ALB: albumin; CHOL: cholesterol; TG: triglyceride; HDL-C: high-density lipoprotein cholesterol; LDL-C: low-density lipoprotein cholesterol; GLB: globulin; A/G: albumin/globulin ratio; AG: anion gap; OSM: osmolality; eGFR: estimated glomerular filtration rate; PT: prothrombin time; APTT: activated partial thromboplastin time; TT: thrombin time; FIB: fibrinogen; PT%: prothrombin time percentage; D-dimer: D-dimer; CRP: C-reactive protein; hs-CRP: high-sensitivity C-reactive protein; NLR: neutrophil-to-lymphocyte ratio; PLR: platelet-to-lymphocyte ratio; MLR: monocyte-to-lymphocyte ratio; SIRI: systemic inflammation response index; SII: systemic immune-inflammation index.

# Supplemental Table S3. Stability of coefficients for selected predictors of stroke-associated pneumonia across stratified 5-fold cross-validation.

| Feature | Fold 1 | Fold 2 | Fold 3 | Fold 4 | Fold 5 | Status |
| --- | --- | --- | --- | --- | --- | --- |
| Selected Features |  |  |  |  |  | Stable |
| NIHSS | 0.154 | 0.148 | 0.161 | 0.150 | 0.155 | Selected |
| WBC | 0.082 | 0.075 | 0.091 | 0.068 | 0.088 | Selected |
| HGB | -0.045 | -0.051 | -0.039 | -0.042 | -0.048 | Selected |
| MCHC | -0.033 | -0.028 | -0.035 | -0.031 | -0.029 | Selected |
| PLT | 0.012 | 0.015 | 0.011 | 0.018 | 0.014 | Selected |
| hs-CRP | 0.210 | 0.198 | 0.205 | 0.215 | 0.201 | Selected |
| ALB | -0.125 | -0.118 | -0.130 | -0.122 | -0.128 | Selected |
| FIB | 0.065 | 0.071 | 0.059 | 0.062 | 0.068 | Selected |
| D-dimer | 0.098 | 0.102 | 0.095 | 0.110 | 0.091 | Selected |
| NLR | 0.112 | 0.105 | 0.118 | 0.109 | 0.115 | Selected |
| SII | 0.135 | 0.142 | 0.128 | 0.130 | 0.145 | Selected |
| mGPS | 0.245 | 0.230 | 0.251 | 0.238 | 0.242 | Selected |
| Dysphagia | 0.178 | 0.185 | 0.170 | 0.165 | 0.180 | Selected |
| COPD | 0.055 | 0.048 | 0.062 | 0.051 | 0.059 | Selected |
| Smoking History | -0.078 | -0.085 | -0.065 | -0.070 | -0.080 | Selected |
| Stroke Subtype (TOAST) | 0.092 | 0.088 | 0.095 | 0.090 | 0.098 | Selected |
| A2DS2 | 0.288 | 0.275 | 0.295 | 0.280 | 0.290 | Selected |
| Age | 0.019 | 0.012 | 0.127 | 0.009 | 0.005 | Selected |
| Excluded Features |  |  |  |  |  | Unstable |
| RBC | 0 | 0 | 0 | 0 | 0 | Excluded |
| HCT | 0.021 | 0 | 0.015 | 0 | 0.011 | Excluded |
| MCV | 0 | 0 | 0 | 0.003 | 0 | Excluded |
| MCH | 0.005 | 0 | 0 | 0 | 0 | Excluded |
| RDW-SD | 0 | 0 | 0 | 0 | 0 | Excluded |
| RDW-CV | 0.018 | 0 | 0 | 0.012 | 0 | Excluded |
| PCT | 0 | 0 | 0 | 0 | 0 | Excluded |
| NEUT | 0.045 | 0 | 0.032 | 0 | 0 | Excluded |
| LYMPH | -0.022 | -0.015 | 0 | 0 | -0.010 | Excluded |
| MONO | 0 | 0 | 0 | 0 | 0 | Excluded |
| BASO | 0 | 0 | 0 | 0 | 0 | Excluded |
| NEUT% | 0 | 0 | 0 | 0 | 0 | Excluded |
| LYMPH% | -0.011 | 0 | 0 | -0.005 | 0 | Excluded |
| CRP | 0.055 | 0.042 | 0 | 0.038 | 0.045 | Excluded |
| TP | 0 | 0 | 0 | 0 | 0 | Excluded |
| GLB | 0.015 | 0 | 0 | 0 | 0 | Excluded |
| A/G | 0 | 0 | 0 | 0 | 0 | Excluded |
| TT | 0.008 | 0 | 0.005 | 0 | 0 | Excluded |
| PLR | 0.065 | 0 | 0.058 | 0.042 | 0 | Excluded |
| MLR | 0 | 0 | 0 | 0 | 0 | Excluded |
| SIRI | 0.088 | 0.075 | 0 | 0.082 | 0.079 | Excluded |
| Sex | 0 | 0 | 0 | 0 | 0 | Excluded |
| HTN | 0.025 | 0 | 0 | 0.018 | 0 | Excluded |
| DM | 0 | 0.035 | 0.028 | 0 | 0 | Excluded |
| HLD | 0 | 0 | 0 | 0 | 0 | Excluded |
| AF | 0.042 | 0.038 | 0 | 0.035 | 0.040 | Excluded |
| Prior Stroke | 0 | 0 | 0 | 0 | 0 | Excluded |
| Alcohol Consumption | -0.015 | 0 | -0.010 | 0 | 0 | Excluded |

Note: COPD: chronic obstructive pulmonary disease; HTN: Hypertension; DM: Diabetes Mellitus; HLD: Hyperlipidemia; AF: Atrial Fibrillation; NIHSS: National Institutes of Health Stroke Scale; A2DS2: Age: Atrial fibrillation: Dysphagia: Sex: Stroke Severity; TOAST: Trial of Org 10172 in Acute Stroke Treatment; mGPS: modified Glasgow Prognostic Score; WBC: white blood cell; RBC: red blood cell; HGB: Hemoglobin; HCT: Hematocrit; MCV: mean corpuscular volume; MCH: mean corpuscular hemoglobin; MCHC: mean corpuscular hemoglobin concentration; PLT: Platelets; RDW-SD: red blood cell distribution width-standard deviation; RDW-CV: red blood cell distribution width-coefficient of variation; PCT: plateletcrit; NEUT: neutrophil count; LYMPH: lymphocyte count; MONO: monocyte count; BASO: basophil count; NEUT%: neutrophil percentage; LYMPH%: lymphocyte percentage; ALB: albumin; TP: total protein; GLB: globulin; A/G: albumin/globulin ratio; TT: thrombin time; FIB: fibrinogen; CRP: C-reactive protein; hs-CRP: high-sensitivity C-reactive protein; NLR: neutrophil-to-lymphocyte ratio; PLR: platelet-to-lymphocyte ratio; MLR: monocyte-to-lymphocyte ratio; SIRI: systemic inflammation response index; SII: systemic immune-inflammation index.

# **Supplemental Table S4. Multicollinearity Diagnostic for Candidate Predictors Based on Variance Inflation Factor (VIF).**

| Variable | VIF Value | Final VIF (Finalized Set) | Status | Interpretation |
| --- | --- | --- | --- | --- |
| A2DS2 Score | 7.42 | -- | Excluded | Severe Multicollinearity |
| NIHSS | 4.15 | 2.62 | Retained | Acceptable |
| Age | 3.56 | 1.54 | Retained | Acceptable |
| Dysphagia | 2.67 | 1.41 | Retained | Acceptable |
| hs-CRP | 2.31 | 2.28 | Retained | Acceptable |
| NLR | 2.44 | 2.41 | Retained | Acceptable |
| SII | 2.51 | 2.49 | Retained | Acceptable |
| WBC | 2.38 | 2.35 | Retained | Acceptable |
| FIB | 2.08 | 2.05 | Retained | Acceptable |
| D-dimer | 2.12 | 2.1 | Retained | Acceptable |
| ALB | 2.19 | 2.16 | Retained | Acceptable |
| HGB | 2.21 | 2.19 | Retained | Acceptable |
| PLT | 1.87 | 1.85 | Retained | Acceptable |
| mGPS | 1.95 | 1.94 | Retained | Acceptable |
| MCHC | 1.55 | 1.53 | Retained | Acceptable |
| Smoking history | 1.42 | 1.41 | Retained | Acceptable |
| Stroke subtype | 1.34 | 1.33 | Retained | Acceptable |
| COPD | 1.18 | 1.17 | Retained | Acceptable |

# Note: COPD: chronic obstructive pulmonary disease; NIHSS: National Institutes of Health Stroke Scale; A2DS2: Age: Atrial fibrillation: Dysphagia: Sex: Stroke Severity; mGPS: modified Glasgow Prognostic Score; WBC: white blood cell; HGB: Hemoglobin; MCHC: mean corpuscular hemoglobin concentration; PLT: Platelets; ALB: albumin; FIB: fibrinogen; hs-CRP: high-sensitivity C-reactive protein; NLR: neutrophil-to-lymphocyte ratio; SII: systemic immune-inflammation index.

# Supplemental Table S5. Pairwise comparisons of the AUC between nine machine learning models using the DeLong test.

| Model | XGBoost | LightGBM | RF | AdaBoost | DT | GBDT | GNB | SVM | KNN |
| --- | --- | --- | --- | --- | --- | --- | --- | --- | --- |
| XGBoost | — | 0.001* | 0.594 | 0.298 | 0.100 | 0.326 | 0.244 | 0.020 | 0.168 |
| LightGBM | — | — | 0* | 0.038 | 0.160 | 0.038 | 0.244 | 0.370 | 0.116 |
| RF | — | — | — | 0.293 | 0.046 | 0.215 | 0.242 | 0.010 | 0.077 |
| AdaBoost | — | — | — | — | 0.396 | 0.580 | 0.382 | 0.014 | 0.568 |
| DT | — | — | — | — | — | 0.590 | 0.304 | 0.227 | 0.497 |
| GBDT | — | — | — | — | — | — | 0.332 | 0.073 | 0.500 |
| GNB | — | — | — | — | — | — | — | 0.046 | 0.444 |
| SVM | — | — | — | — | — | — | — | — | 0.137 |
| KNN | — | — | — | — | — | — | — | — | — |

Note: RF: Random Forest; XGBoost: Extreme Gradient Boosting; LightGBM: Light Gradient Boosting Machine; AdaBoost: Adaptive Boosting; GBDT: Gradient Boosting Decision Tree; DT: Decision Tree; GNB: Gaussian Naive Bayes; KNN: K-Nearest Neighbors; SVM: Support Vector Machine; LightGBM: Light Gradient Boosting Machine.

Statistical Note: *p* values were derived from the *Z*-scores of the two-tailed DeLong test.

* indicates statistical significance (*p* < 0.0056).

A *p* value > 0.0056 indicates no significant difference between the AUCs of the two compared models.

# Supplemental Table S6. Sensitivity Analysis of Missing Data Handling.

| Cohort | Metric | Primary Model (MissForest Imputed) | Sensitivity Model (Complete Case Analysis) | *p* value |
| --- | --- | --- | --- | --- |
| Internal Test Set | AUC (95% CI) | 0.898 (0.845–0.951) | 0.879 (0.816–0.942) | 0.284 |
|  | Accuracy | 0.822 | 0.798 |  |
|  | Sensitivity | 0.782 | 0.745 |  |
|  | Specificity | 0.851 | 0.835 |  |
|  | *F1*-score | 0.801 | 0.772 |  |
|  | Brier Score | 0.214 (0.209–0.220) | 0.226 (0.218–0.235) |  |
| Temporal Validation | AUC (95% CI) | 0.875 (0.841–0.910) | 0.856 (0.812–0.900) | 0.231 |
|  | Accuracy | 0.8 | 0.779 |  |
|  | Sensitivity | 0.792 | 0.75 |  |
|  | Specificity | 0.814 | 0.797 |  |
|  | *F1*-score | 0.831 | 0.803 |  |
|  | Brier Score | 0.214 (0.209–0.220) | 0.228 (0.219–0.237) |  |

# Note: AUC: Area Under the Receiver Operating Characteristic Curve; CI: Confidence Interval.

# **Supplemental Table S7. Baseline Characteristics and Predictors Across Cohorts.**

| Characteristics | Derivation Cohort (n=1,123) | Temporal Validation Cohort (n=199) | *p* value*  (vs. Derivation) | External Validation Cohort (n=287) | *p* value^†^  (vs. Derivation) |
| --- | --- | --- | --- | --- | --- |
| Outcome |  |  |  |  |  |
| SAP, n (%) | 237 (21.1) | 38 (19.1) | 0.503 | 74 (25.8) | 0.086 |
| Demographics |  |  |  |  |  |
| Age, years, median (IQR) | 69 (61–77) | 68 (60–76) | 0.452 | 75(67–82) | <0.001 |
| Smoking History, n (%) | 378 (33.6) | 64 (32.2) | 0.695 | 100 (34.8) | 0.702 |
| Sex , n (%) (Male) | 714 (63.6) | 125 (62.8) | 0.824 | 185 (64.5) | 0.776 |
| Clinical Assessment |  |  |  |  |  |
| NIHSS, median (IQR) | 3 (1–6) | 3 (1–5) | 0.912 | 5 (2–9) | 0.043 |
| A2DS2, median (IQR) | 2 (1–4) | 2 (1–4) | 0.885 | 2 (1–4) | 0.962 |
| Dysphagia, n (%) | 110 (9.8) | 18 (9.0) | 0.745 | 73 (25.4) | <0.001 |
| Stroke Subtype (TOAST), n (%) |  |  | 0.251 |  | 0.518 |
| LAA | 733 (65.27%) | 127 (63.82) |  | 188 (65.51) |  |
| CE | 158 (14.07%) | 27 (13.57) |  | 41 (14.29) |  |
| SAO | 179 (15.94%) | 34 (17.09) |  | 44 (15.33) |  |
| SUE | 37 (3.29%) | 4 (2.01) |  | 13 (4.53) |  |
| SOE | 16 (1.42%) | 7(3.52) |  | 1(0.35) |  |
| Comorbidities |  |  |  |  |  |
| COPD, n (%) | 73 (6.50) | 12 (6.03) | 0.789 | 19 (6.62) | 0.943 |
| HTN, n (%) | 920 (81.92) | 161 (80.90) | 0.725 | 236 (82.29) | 0.905 |
| DM, n (%) | 451 (40.16) | 83 (41.71) | 0.672 | 158 (55.05) | <0.001 |
| AF, n (%) | 50 (4.45) | 10 (5.03) | 0.715 | 11 (3.83) | 0.648 |
| Novel Inflammatory Indices |  |  |  |  |  |
| mGPS, n (%) |  |  | 0.992 |  | 0.987 |
| 0 | 1030 (91.72%) | 182 (91.5) |  | 264 (92.0) |  |
| 1 | 60 (5.34%) | 11 (5.5) |  | 15 (5.2) |  |
| 2 | 33 (2.94%) | 6 (3.0) |  | 8 (2.8) |  |
| SII, median (IQR) | 545 (365–794) | 538 (370–785) | 0.765 | 550 (360–801) | 0.812 |
| NLR, median (IQR) | 2.56 (1.80–3.64) | 2.51 (1.82–3.60) | 0.654 | 2.60 (1.78–3.70) | 0.589 |
| Laboratory Findings |  |  |  |  |  |
| hs-CRP, mg/L, median (IQR) | 0.69 (0.50–3.40) | 0.72 (0.51–3.45) | 0.512 | 0.85 (0.55–4.10) | 0.048 |
| WBC , ×10⁹/L, median (IQR) | 6.81 (5.73–8.18) | 6.75 (5.65–8.15) | 0.745 | 7.10 (5.90–8.45) | 0.065 |
| PLT, ×10⁹/L, median (IQR) | 215 (178–253) | 210 (175–250) | 0.456 | 218 (182–255) | 0.398 |
| HGB, g/L, median (IQR) | 135 (124–146) | 133 (122–145) | 0.387 | 136 (126–147) | 0.412 |
| MCHC, g/L, median (IQR) | 326 (315–338) | 325 (313–337) | 0.565 | 327 (316–339) | 0.621 |
| ALB, g/L, median (IQR) | 39.0 (36.2–41.6) | 38.9 (36.0–41.5) | 0.789 | 39.1 (36.5–41.8) | 0.645 |
| FIB, g/L, median (IQR) | 2.83 (2.38–3.35) | 2.80 (2.35–3.30) | 0.543 | 2.85 (2.40–3.38) | 0.612 |
| D-dimer, mg/L, median (IQR) | 0.45 (0.24–1.41) | 0.44 (0.23–1.38) | 0.812 | 0.46 (0.25–1.45) | 0.765 |

Note: SAP: stroke-associated pneumonia; IQR: interquartile range; COPD: chronic obstructive pulmonary disease; NIHSS: National Institutes of Health Stroke Scale; A2DS2: Age: Atrial fibrillation: Dysphagia: Sex: Stroke Severity score; TOAST: Trial of Org 10172 in Acute Stroke Treatment; LAA: Large artery atherosclerosis; CE: Cardioembolism; SAO: Small artery occlusion; SUE: Stroke of Undetermined Etiology; SOE: Stroke of other determined etiology; mGPS: modified Glasgow Prognostic Score; SII: systemic immune-inflammation index; NLR: neutrophil-to-lymphocyte ratio; WBC: white blood cell; MCHC: mean corpuscular hemoglobin concentration; hs-CRP: high-sensitivity C-reactive protein; LAA: Large-artery atherosclerosis; CE: Cardioembolism; SAO: Small-vessel occlusion; HTN: Hypertension; DM: Diabetes Mellitus; AF: Atrial Fibrillation; PLT: Platelets; HGB: Hemoglobin; ALB: albumin; FIB: fibrinogen.

Statistical Statement:

**p* values indicate the difference between the Temporal Validation Cohort and the Derivation Cohort.

†*p* values indicate the difference between the External Validation Cohort and the Derivation Cohort.

Comparisons were performed using the Mann-Whitney U test for continuous variables and the Chi-square test or Fisher's exact test for categorical variables.
